# Rapid and Reversible Lithium Insertion in the Wadsley–Roth-Derived Phase NaNb_13_O_33_

**DOI:** 10.1021/acs.chemmater.3c01066

**Published:** 2023-08-02

**Authors:** Ashlea R. Patterson, Rodrigo Elizalde-Segovia, Kira E. Wyckoff, Arava Zohar, Patrick P. Ding, Wiley M. Turner, Kenneth R. Poeppelmeier, Sri R. Narayan, Raphaële J. Clément, Ram Seshadri, Kent J. Griffith

**Affiliations:** † Materials Department and Materials Research Laboratory, University of California, Santa Barbara, Santa Barbara, California 93106, United States; ‡ Department of Chemistry, University of Southern California, Los Angeles, California 90007, United States; § California NanoSystems Institute, University of California, Santa Barbara, Santa Barbara, California 93106, United States; ∥ Department of Chemistry, Northwestern University, Evanston, Illinois 60208, United States; ⊥ Department of Materials Science and Engineering, Northwestern University, Evanston, Illinois 60208, United States

## Abstract

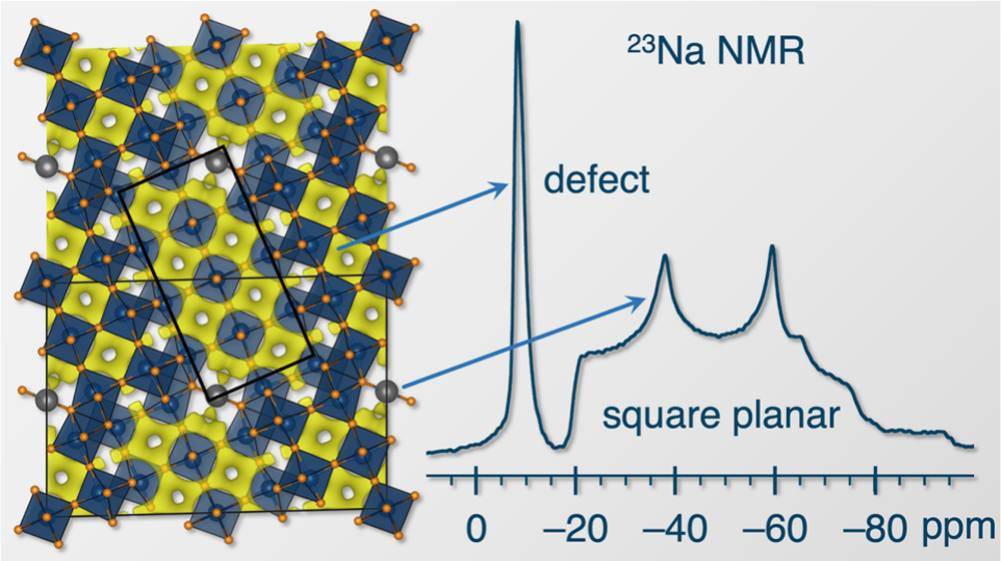

The development of new high-performing battery materials is critical for meeting the energy storage requirements of portable electronics and electrified transportation applications. Owing to their exceptionally high rate capabilities, high volumetric capacities, and long cycle lives, Wadsley–Roth compounds are promising anode materials for fast-charging and high-power lithium-ion batteries. Here, we present a study of the Wadsley–Roth-derived NaNb_13_O_33_ phase and examine its structure and lithium insertion behavior. Structural insights from combined neutron and synchrotron diffraction as well as solid-state nuclear magnetic resonance (NMR) are presented. Solid-state NMR, in conjunction with neutron diffraction, reveals the presence of sodium ions in perovskite A-site-like block interior sites as well as square-planar block corner sites. Through combined experimental and computational studies, the high rate performance of this anode material is demonstrated and rationalized. A gravimetric capacity of 225 mA h g^–1^, indicating multielectron redox of Nb, is accessible at slow cycling rates. At a high rate, 100 mA h g^–1^ of capacity is accessible in 3 min for micrometer-scale particles. Bond-valence mapping suggests that this high-rate performance stems from fast multichannel lithium diffusion involving octahedral block interior sites. Differential capacity analysis is used to identify optimal cycling rates for long-term performance, and an 80% capacity retention is achieved over 600 cycles with 30 min charging and discharging intervals. These initial results place NaNb_13_O_33_ within the ranks of promising new high-rate lithium-ion battery anode materials that warrant further research.

## Introduction

As we transition from fossil fuels to low-carbon energy sources, faster charging, higher energy, and higher power lithium-ion batteries (LIBs) are required to support widespread electrification. LIBs typically use graphitic carbon as the anode material given its high lithium storage capacity, low cost, and low voltage vs Li^+^/Li, which leads to a high energy density when paired with a 4-V-class cathode material such as Li(Ni,Mn,Co,Al)O_2_.^1,2^ However, the low voltage of graphite lithiation relative to the lithium metal deposition potential can lead to electrolytic side reactions and lithium metal plating, the results of which are increased impedance, shortened battery lifetimes, and thermal runaway should lithium dendrites traverse the separator.^3−5^ These issues, which are especially problematic at high current densities, have motivated the search for alternative anode materials that operate between 1.0 and 2.0 V vs Li^+^/Li.^6,7^

Wadsley–Roth compounds are a family of (mostly) transition metal oxides that have garnered interest as fast-charging LIB anodes. Many of these compounds were first reported in the 1950s–1960s^8−13^ but were not considered as electrode materials until the seminal work of Cava et al.^14−16^ Research into Wadsley–Roth electrodes declined until two 2011 papers by Han, Goodenough, and colleagues demonstrated excellent rate performance, respectable capacities, and stable cycling with the Wadsley–Roth phase TiNb_2_O_7_ as a lithium intercalation anode.^6,7^ Wadsley–Roth compounds typically operate above 0.8 V vs Li^+^/Li and are thus not subject to lithium plating or deleterious side reactions with the electrolyte. They also tend to insert more than one lithium per transition metal with a minor volume expansion. This allows for large and reversible lithium storage capacities and good long-term electrochemomechanical stability. Finally, Wadsley–Roth anodes have demonstrated excellent high-rate performance.^6,7,17−21^

Wadsley–Roth compounds are characterized by *m* × *n* blocks of corner-sharing MO_6_ octahedra joined by edge-sharing octahedra in “shear” planes, as first described by Roth in 1965.^9,10^ Unlike one-dimensional shear structures and layered vacancy-ordered Wadsley–Roth derivatives that exhibit relatively poor capacity retention upon electrochemical cycling,^22−24^ Wadsley–Roth phases exhibit good stability upon repeated lithium (de)intercalation because of the structural rigidity and minimal volume expansion imparted by the shear planes.^18,25−28^ Moreover, a combination of experimental and computational studies on Wadsley–Roth phases have provided evidence for rapid, quasi-one-dimensional lithium diffusion down parallel octahedral block channels paired with facile interchannel hopping, which helps to redistribute lithium and prevent blockages.^26,29,30^ This combination of long-range diffusion and short-range redistribution accounts for the extremely high lithium diffusion coefficients reported for Wadsley–Roth electrodes, which are on par with those of lithium-conducting solid electrolytes.^18,19,31^ Additional studies have shown good electronic conductivity in Wadsley–Roth phases once they are partially reduced by either oxygen loss or lithium insertion.^14,29,32−34^ This rapid intercalation behavior has been observed in micrometer-scale particles, indicating that Wadsley–Roth phases do not require nanostructuring for high-rate performance.

**Figure 1:**
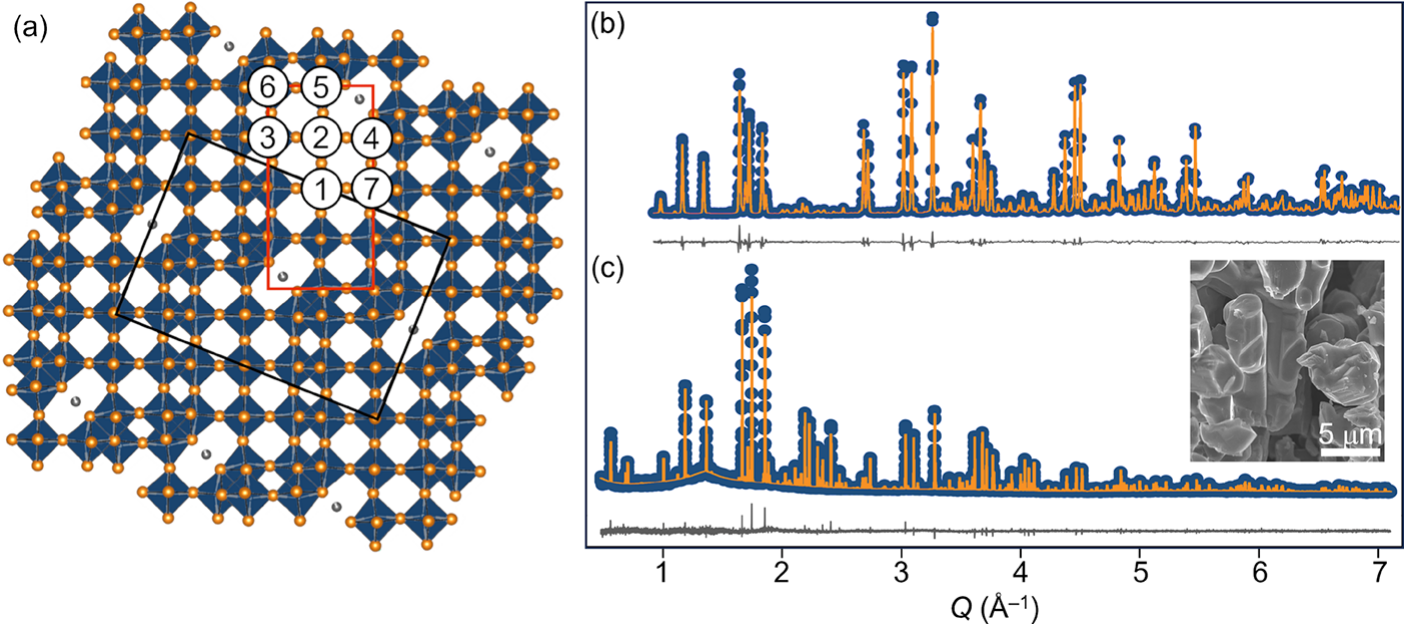
(a) Schematic of the NaNb_13_O_33_ crystal structure highlighting the (5×3–2)_*∞*_ blocks of NbO_6_ octahedra and each unique Nb site. (b) Neutron powder diffraction of 1-NNO obtained at 300 K, λ_c_ = 1.5 Å. (c) Synchrotron X-ray powder diffraction of 1-NNO at 300 K, λ_c_ = 0.45788 Å. The diffuse background feature is due to the Kapton capillary sample holder. The inset shows an SEM image of pristine 1-NNO particles

The present study comprises an in-depth structural and initial electrochemical investigation of the Wadsley–Roth-like phase NaNb_13_O_33_. The structure of this composition within the Nb_2_O_5_–Na_2_O solid solution system was first reported in 1965.^35^ Its block-defect structure and sodium conductivity were investigated in 1984,^36^ and its thermodynamic stability has been the subject of several reports.^37−39^ This compound deviates from the typical Wadsley–Roth structure in that transition-metal-centered octahedra are missing from the corners of the blocks, and instead, sodium ions occupy square planar sites. This is a relatively rare Na^+^ coordination that results in large rectangular tunnels connecting the Na sites. Following the (*m* × *n*)_*∞*_ Wadsley–Roth nomenclature where *m* and *n* are the block dimensions in units of octahedra and the subscript denotes the connectivity of the blocks in the block plane, we propose that the deviation in NaNb_13_O_33_ should be denoted (5×3–2)_*∞*_ (Figure 1a).

We report structural insights from combined neutron and synchrotron diffraction of NaNb_13_O_33_ along with ^23^Na solid-state nuclear magnetic resonance spectroscopy. A new picture of the atomic-scale defect structure emerges with sodium cations occupying not only the square-planar sites but also perovskite-like sites within the block interiors. Density functional theory calculations of the electronic structure and bond-valence sum mapping of the electrostatic landscape suggest good electronic and ionic conductivity. This is confirmed by the electrochemical behavior of this material in Li half-cell batteries, which reveal excellent rate capability and long-term cyclability with micrometer-sized NaNb_13_O_33_ particles. Finally, galvanostatic cycling suggests Nb multielectron redox, which enables large amounts of lithium to be stored in this promising new anode material.

## Methods

### Solid-State Synthesis of NaNb_13_O_33_

NaNb_13_O_33_ was prepared by multiple independent solid-state methods. In the first preparation method, resulting in the sample hereafter termed 1-NNO, Nb_2_O_5_ (CBMM, 99.8%, white powder, 1:1 T-phase:H-phase) and Na_2_CO_3_ (Sigma, 99.95–100.05%, anhydrous, white powder, dried at 250 °C for 8 h) were hand-mixed and ground in an agate mortar and pestle for 10 min before being pressed into two 5 g, 25.4 mm diameter pellets at 300 MPa. The pellets were placed in a Pt crucible and fired in a three-stage heating program, all with 5 °C min^–1^ heating rates, at 400 °C for 6 h, 820 °C for 12 h, and 1150 °C for 18 h before being cooled in the furnace under zero power. Note that the sample prepared for synchrotron diffraction used a single heating step at 1150 °C for 24 h.

In the second solid-state preparation method, which resulted in the sample termed 2-NNO, NaNbO_3_ was first prepared by hand-grinding a 1:1 molar ratio of Nb_2_O_5_ (Materion, ≥99%) and Na_2_CO_3_ (Sigma, ≥99%) in an agate mortar and pestle for 20 min. The resulting mixture was pressed into 700 mg, 13 mm pellets under 3.5 t of pressure. Pellets were placed on a bed of sacrificial powder within an alumina crucible, heated to 1100 °C at a rate of 10 °C min^–1^, sintered for 12 h, and then air-quenched. The same grinding, pelletizing, heating, and quenching procedure was then repeated to produce NaNb_13_O_33_ from a hand-ground mixture of NaNbO_3_ and Nb_2_O_5_ in a 1:6 molar ratio.

### Scanning Electron Microscopy (SEM)

To characterize the morphology and size of 1- and 2-NNO particles, NaNb_13_O_33_ pellets were hand-ground for approximately 10 min in an agate mortar and pestle. The powdered samples were then pressed onto double-sided carbon tape and measured with a Hitachi S4800 (1-NNO) or an Apreo C FEG (ThermoFisher) (2-NNO) microscope. SEM images were collected using secondary electron (SE) detection with 10 kV accelerating voltage.

### Diffraction

Synchrotron X-ray powder diffraction (SXRPD) data were collected on both 1- and 2-NNO at 300 K on the 11-BM beamline at the Advanced Photon Source at Argonne National Laboratory with a wavelength λ = 0.45788 Å. Time-of-flight neutron powder diffraction (TOF-NPD) data of 1-NNO were obtained on the POWGEN diffractometer at the Spallation Neutron Source (SNS) located at Oak Ridge National Laboratory.^40^ Approximately 10 g of NaNb_13_O_33_ was loaded into an 8 mm diameter cylindrical vanadium can. Data were collected on POWGEN detector bank 2 with a center wavelength of 1.5 Å at 20 and 300 K with scan times of 75 and 95 min, respectively, and detector bank 3 with a center wavelength of 2.665 Å at 300 K for 5 min. Combined Rietveld analysis on SXRPD and TOF-NPD data was performed using GSAS-II.^41^ Fitting information and refined patterns of both samples are included in the Supporting Information (S1–S2).

### Nuclear Magnetic Resonance (NMR)

To better understand the local structure of as-prepared NaNb_13_O_33_, magic-angle spinning (MAS) solid-state ^23^Na NMR was performed on 1- and 2-NNO compounds at room temperature.

Solid-state ^23^Na MAS NMR spectra were acquired on 1-NNO using a Bruker AVANCE III 400 MHz (9.4 T) spectrometer and a 1.6 mm Phoenix HX MAS probe or a 4 mm Bruker HX MAS probe. The 1-NNO sample was packed into zirconia rotors in air and rotated about the magic angle at 12.5 kHz to obtain high signal-to-noise spectra using a 4 mm rotor and at 38 kHz for the variable-temperature study using a 1.6 mm rotor. One-pulse spectra were collected using an rf pulse of either 0.8 μs (1.6 mm probe) or 1.3 μs (4 mm probe), corresponding to half the length of a liquid-state ^23^Na π/2 pulse to account for the doubling of the nutation frequency of this spin-3/2 nucleus in the solid state. *T*_1_ values were measured with a saturation–recovery pulse sequence, and recycle delays were subsequently set to 5 × *T*_1_. For comparison, spectra of NaNbO_3_ and Na_13_Nb_35_O_94_ were obtained using similar data acquisition parameters. ^23^Na shifts of 1-NNO, NaNbO_3_, and Na_13_Nb_35_O_94_ were referenced to aqueous 1.0 M NaCl at 0 ppm. Actual sample temperatures during variable-temperature MAS NMR data acquisition differ from the value measured by the thermocouple due to frictional heating of the sample. The sample temperature was externally calibrated by leveraging the temperature dependence of the ^207^Pb shift of a lead nitrate reference sample.^42,43^ A solid-state ^23^Na MAS NMR spectrum of the 2-NNO material was also acquired, as detailed in the Supporting Information.

### Electronic Structure and Bond Valence Sum Calculations

To understand the electronic properties of the host structure, density functional theory (DFT) calculations were performed using the Vienna Ab initio Simulation Project (VASP)^44−46^ code, projector augmented-wave (PAW) pseudopotentials,^47,48^ and the general-gradient-approximation Perdew–Burke–Ernzerhof (PBE)^49^ functional. Structural models were obtained from a joint refinement of 1-NNO neutron and synchrotron XRD diffraction patterns obtained at 20 K. Lattice parameters and atomic positions were optimized by using a plane-wave energy cutoff of 500 eV and a *k*-point mesh with a length-density parameter of *R*_*k*_ = 25 Å, corresponding to a 7 × 2 × 2 Γ-centered mesh. Structural relaxation was followed by static calculations of the projected density of states (DOS) and electronic band structure, which was postprocessed using the Sumo package.^50^

To estimate the pathways for ion intercalation in the three-dimensional NaNb_13_O_33_ host structure, bond valence sum mapping was performed with either Li^+^ or Na^+^ as the mobile ion using softBV-GUI ver. 1.2.7.^51−53^ Energy barriers for ion hopping within the NaNb_13_O_33_ unit cell were visualized in VESTA^54^ using an isosurface energy cutoff of 0.1 valence units.

Finally, DFT, as implemented in plane wave code CASTEP v19.11, was used to compute the quadrupolar tensor of NaNb_13_O_33_. The calculations were performed using “on-the-fly” ultrasoft pseudopotentials and the PBE exchange–correlation functional.^49,55−57^ The plane-wave basis set was truncated at an energy cutoff of 700 eV, and integration over reciprocal space was performed using a 2 × 7 × 2 Monkhorst–Pack grid.^58^ Structures were geometry optimized prior to NMR calculations.^59−61^ Spectral simulations of the calculated quadrupolar tensor were performed with the Solid Lineshape Analysis (SOLA) tool in TopSpin v4.0.9.

### Electrochemical Characterization

Electrodes composed of 2-NNO, Super P carbon black, and poly(vinylidene difluoride) (PVDF) were fabricated in a weight ratio of 80:10:10. Pristine 2-NNO was blended with carbon black (MTI Super P) in a mortar and pestle and mixed into a solution of PVDF (MTI) in *N*-methyl-2-pyrrolidone (NMP, Sigma-Aldrich, 40 g L^–1^) to produce a slurry. The slurry was cast onto copper foil using a doctor blade, giving a coating thickness of approximately 40 μm. The resulting film was vacuum-dried at 80 °C overnight and punched into 14 mm diameter discs, resulting in approximate active material mass loadings of 2.0–5.5 mg cm^–2^. The electrodes were incorporated into CR2032 coin cells from MTI in an argon-filled glovebox, with polished lithium metal (MTI) as a counter electrode and glass fiber separators (Whatman GF/C). An electrolyte of 1.0 M lithium bis(trifluoromethanesulfonyl)imide (LiTFSI, Sigma-Aldrich) dissolved in 1:1 v/v ethylene carbonate (EC, Sigma-Aldrich) and dimethyl carbonate (DMC, Sigma-Aldrich) was added to the cell in a ratio of 25 μL of electrolyte per milligram of active material. Half-cells of 2-NNO electrodes vs sodium metal were fabricated in a similar manner, as detailed in the Supporting Information.

On the basis of reduction of Nb^5+^ to Nb^4+^, 13 Li or Na ions inserted into NaNb_13_O_33_ would yield a theoretical capacity of 198 mA h g^–1^ based on the molecular mass of the pristine compound as is standard for anode materials. Cycling rate is defined as C/*n*, where *n* is the number of hours to complete a single charge or discharge. Therefore, a C/10 rate corresponds to the current density needed to fully charge or discharge to the theoretical capacity in 10 h or a current density of 19.8 mA g^–1^. All electrochemical experiments were performed using Biologic potentiostats.

## Results and Discussion

### Characterization of the NaNb_13_O_33_ Host Structure

Scanning electron microscopy (SEM) images of pristine 1-NNO show particles on the order of 5–50 μm, while SEM images of 2-NNO show particles on the order of 1–10 μm (Figure 1 inset and Figure S4). Synchrotron powder X-ray diffraction (SXPRD) and neutron powder diffraction (NPD) patterns were collected and jointly refined to examine phase purity, octahedral distortions, and sodium position(s) and occupancy(ies) in NaNb_13_O_33_. X-ray diffraction confirms the phase purity of the NaNb_13_O_33_ samples prepared using the two synthesis methods (Figures 1c and S2). Neutron diffraction gives atomic displacement parameters (ADPs) for each Nb and Na site within the structure at 20 and 300 K (Figure 1b). Square-planar Na shows a relatively large atomic displacement parameter (ADP) at room temperature, indicating greater motion of the Na^+^ ions around their nominal positions in the crystal structure. ADPs and second-order Jahn–Teller distortion parameters for the NbO_6_ octahedra are listed in Table S1.

**Figure 2:**
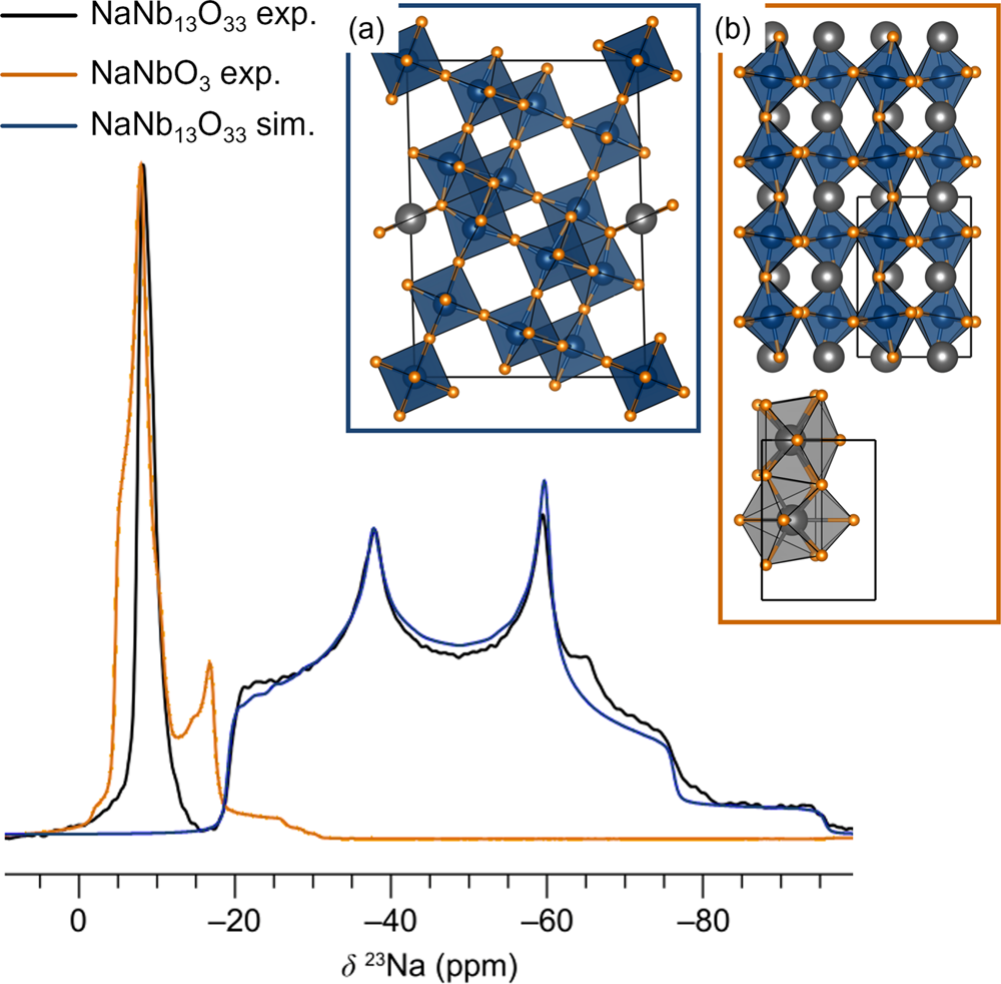
^23^Na MAS NMR of 1-NNO featuring two distinct resonances (black curve): a fit of the quadrupolar environment with a center of mass at approximately −50 ppm (blue curve) and an overlaid experimental spectrum of NaNbO_3_ (orange curve). Spectra were acquired at room temperature at 9.4 T and 12.5 kHz MAS. Box (a) shows the NaNb_13_O_33_ structure with its square-planar Na environment, which is assigned to the strongly quadrupolar line shape, while box (b) shows the Na environments in NaNbO_3_. Grey spheres and polyhedra correspond to sodium, blue octahedra are NbO_6_.

^23^Na solid-state MAS NMR of NaNb_13_O_33_ revealed two environments (Figure 2, black curve): a sharp resonance at −8 ppm and a broad resonance centered at −50 ppm, with respective relative integrated intensities of 16:84. The *T*_1_ relaxation times of the high- and low-frequency components are 3.1 and 0.91 s, respectively. The relaxation difference is sensible given that the low-frequency resonance has a larger quadrupolar interaction, and quadrupolar relaxation is expected to be the dominant relaxation mechanism for ^23^Na in diamagnetic NaNb_13_O_33_. This low-frequency resonance, with an isotropic shift of −15 ppm, a large quadrupolar coupling constant of *C*_*Q*_ = 3.74 MHz, and asymmetry η_*Q*_ = 0.42 (Figure 2, blue curve), corresponds to a spherically asymmetric Na site. It is therefore assigned to the square-planar coordinated Na site in the NaNb_13_O_33_ crystal structure. The quadrupolar parameters measured for this Na site, and particularly the magnitude of the quadrupolar coupling, are in good agreement with results from CASTEP NMR calculations (*C*_*Q*_ = 3.96 MHz; η_*Q*_ = 0.13) on the Na-ordered parent structure.

The sharp Na environment, however, cannot be explained by the Na-ordered pristine NaNb_13_O_33_ structure with its single square-planar Na coordination. Curiously, it is consistent with the presence of Na in the 12-coordinate site within the octahedral block channels (i.e., the perovskite A-site) of this Wadsley–Roth-derived phase. A Na atom occupying this perovskite-like site within NaNb_13_O_33_ would have a mean distance from the 12 nearest atoms of 2.82 Å, which is quite similar to that of NaNbO_3_, and indeed, the chemical shift is virtually identical with that of Na in perovskite NaNbO_3_ (Figure 2, orange curve). However, no secondary phase of NaNbO_3_ was observed in either high-resolution SXRPD or NPD, and the line shape of the sharp resonance in the NaNb_13_O_33_ sample is narrower than that observed in any of the polymorphs of NaNbO_3_.^62,63^ The narrower resonance could be the result of (i) fast local Na dynamics or (ii) a less distorted Na local environment in NaNb_13_O_33_—or perhaps a combination of both.

In NaNbO_3_, the Na sites are highly distorted (Figure 2b).^64^ With a Goldschmidt tolerance factor less than one, NbO_6_ octahedra in NaNbO_3_ may tilt and rotate around the Na sites, while second-order Jahn–Teller distortions associated with Nb^5+^ add to the asymmetry. Conversely, the presence of significant edge-sharing in NaNb_13_O_33_ prevents octahedral rotation and tilting, and the occupancy of sodium on the perovskite A-site position in NaNb_13_O_33_ is far less than unity (vide infra), which will affect electrostatic repulsion. Hence, Na ions in the perovskite-like sites of NaNb_13_O_33_ are expected to be in more symmetrical environments than those in NaNbO_3_.

Overall, there is evidence to suggest that the additional NMR resonance should be attributed to a fraction of Na in the interstitial, perovskite A-site-like positions of NaNb_13_O_33_ rather than an impurity phase such as NaNbO_3_. This idea is supported by further evidence from diffraction. First, in the joint refinement, the occupancy of Na in the square-planar site freely refines to 0.85(4) at 20 K and 0.89(2) at 300 K, which are within error and similar to the 84% integrated NMR signal intensity obtained for the low-frequency component of 1-NNO (87% for 2-NNO (Figure S3)). We note that there are challenges to the precise quantification of NMR signal intensities of sites with significantly different quadrupolar coupling interaction magnitudes.^65^ In the case of a spin-3/2 nucleus such as ^23^Na, 60% of the total intensity is contained in the satellite transitions^66^ that are expected to be distributed over the spinning sidebands for the highly quadrupolar square-planar site but would be expected to be partially or fully folded into the sharp centerband of the perovskite-like site. Thus, a reasonable bound on the relative sodium occupancies from these data sets would be 84%–93% for the broad low-frequency signal and 7%–16% for the narrow high-frequency signal.

Second, Fourier difference maps of the neutron diffraction data at 20 K reveal nuclear density near the center of the perovskite-like sites in NaNb_13_O_33_ (Figure S9). The data are too noisy to yield accurate refined Na occupations or ADPs within each perovskite-like site, which is not surprising given that there is only one Na per NaNb_13_O_33_ formula unit, and there are six such sites that are expected to sum to 7%–16% of the Na, so the occupancies may only be around 1%–2% per site. Neutron diffraction was helpful here because the relative scattering of Na vs Nb is roughly 1:2 with neutrons, compared to 1:4 with X-rays, and because nuclear density is less diffuse than electron density.

The third and final piece of evidence for this assignment of the NMR data, and for the presence of point defects in NaNb_13_O_33_, comes from phase boundary mapping.^67,68^ With this technique, the target compound NaNb_13_O_33_ is purposefully made off-stoichiometric on the Na-poor side and the Na-rich side of the phase diagram until a secondary phase appears. In the case of NaNb_13_O_33_ prepared under the 1-NNO synthesis conditions, the adjacent Na-poor phase is H–Nb_2_O_5_ and the adjacent Na-rich phase is the bronze-like phase Na_13_Nb_35_O_94_. With excess Nb_2_O_5_ added to the synthesis, reflections from H–Nb_2_O_5_ are clearly visible in the XRD pattern, and there is nearly no change in the ^23^Na NMR spectrum (Figures S6 and S7), suggesting that the Na-containing phase is unaffected. With excess Na_2_CO_3_ added to the synthesis, small Bragg reflections from the bronze-like phase become visible in XRD, and significant ^23^Na NMR signal intensity appears around −22 ppm, along with broadening around the base of the signal at −8 ppm (Figure S6). A spectrum of pure Na_13_Nb_35_O_94_ reveals a strong resonance at −22 ppm and a weaker resonance at −8 ppm that is four times broader than the sharp signal at −8 ppm in NaNb_13_O_33_ (Figure S6). The crystal structure of Na_13_Nb_35_O_94_ contains four unique sodium sites, with two in four-sided tunnels, much like the perovskite-like site in NaNb_13_O_33_, and two in pentagonal tunnels that are common in bronze-like compounds. Although it goes beyond the scope of this work, we propose that the resonance at −8 ppm in Na_13_Nb_35_O_94_ originates from the perovskite A-cation-like site, which would be consistent with those of NaNb_13_O_33_ and NaNbO_3_.

Taken together, the structural data show that NaNb_13_O_33_ contains approximately 90% Na on the square-planar site with 10% distributed onto the perovskite A-site-like environments within the (5×3–2)_*∞*_ blocks of NbO_6_ octahedra.

Near-ambient Na dynamics were investigated with variable-temperature ^23^Na NMR (Figure S8). The spectra collected from −13 to 72 °C are nearly temperature-independent. A small drift in the resonance frequency is observed, but no appreciable line-broadening occurs across the temperatures measured. At low temperatures, no additional environments appear, indicating that the Na^+^ ions have not been “frozen out”, and at elevated temperatures, the line widths do not narrow or merge, indicating no appreciable chemical exchange between Na sites. Bovin’s study of NaNb_13_O_33_ reported Na^+^-ion conductivities from 6 × 10^–2^ to 6 × 10^–4^ mS cm^–1^ for temperatures from 450 to 275 °C.^36^ It is therefore unsurprising that significant Na dynamics are not observed over the much lower temperature range investigated here, which is also the relevant temperature range for most Li-ion battery applications.

### Characterization of NaNb_13_O_33_ as a Battery Electrode Material

**Figure 3:**
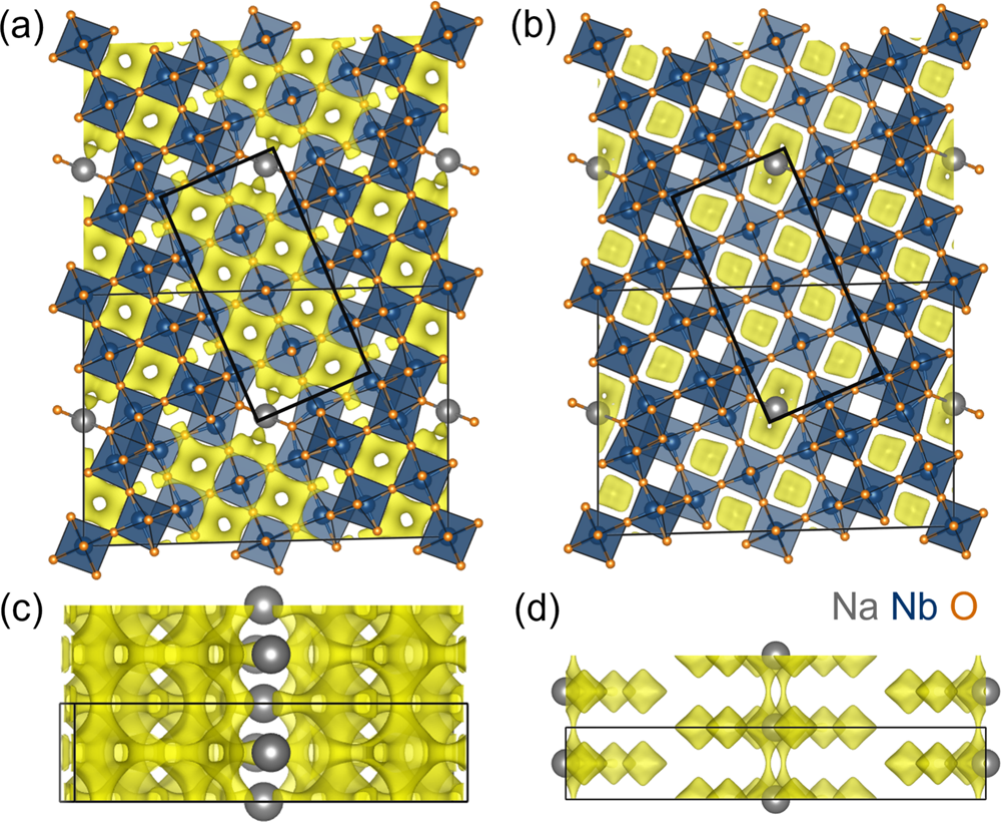
Bond-valence sum energy map of Li^+^ (a, c) and Na^+^ (b, d) as mobile ions in ordered NaNb_13_O_33_ viewed in the *ac* plane (top) and the *ab* plane (bottom). Low-energy-barrier Li-ion conduction pathways (Δ*v* = 0.1 valence units) are shown in yellow.

Bond-valence sum energy (BVSE) mapping was performed on NaNb_13_O_33_ to approximate its Li- and Na-ion diffusion pathways and thus its performance as a host material for Li- and Na-ion battery applications. BVSE is a simple electrostatic method that uses the bond valence site energy approach to estimate the energy barriers relevant to ion diffusion within a crystal structure.^69^ In this method, a mobile cation is chosen (here, Li^+^ or Na^+^), and the interaction energy between this ion and the entire structure, except for other ions of the same type, is calculated over a grid of locations throughout the structure. The resulting array of BVSE energies provides an estimation of relative hopping energy barriers, a representation of the energy landscape for the mobile ion, and a picture of conduction pathways.^21,51−53,70,71^ The energy isosurface for Δ*v* = 0.1 valence units is displayed for NaNb_13_O_33_ with Li^+^ and Na^+^ as mobile ions in Figure 3, where Δ*v* is the half-width (in valence units) of the volume accessible to the mobile ion.^69^ From the extensive Li^+^ conduction pathways visible at this cutoff, BVSE suggests low-energy barriers for intrablock hopping in the *ac* plane as well as low-energy-barrier pathways down the *b* axis within the blocks (Figure 3a,c). This combination of facile 1D diffusion down block channels and intrablock Li redistribution is consistent with previous studies of Wadsley–Roth Li diffusion mechanisms, and it has been credited for their Li diffusion coefficients on par with those of solid electrolyte materials.^18,19,29−31^ Conversely, BVSE does not predict facile Na^+^ hopping within octahedral blocks or down channels (Figure 3b,d). The only predicted low-energy-barrier Na^+^ motion is localized within each NbO_6_ cage and existing square-planar sites, suggesting that NaNb_13_O_33_ would make a poor Na-ion conductor.

**Figure 4:**
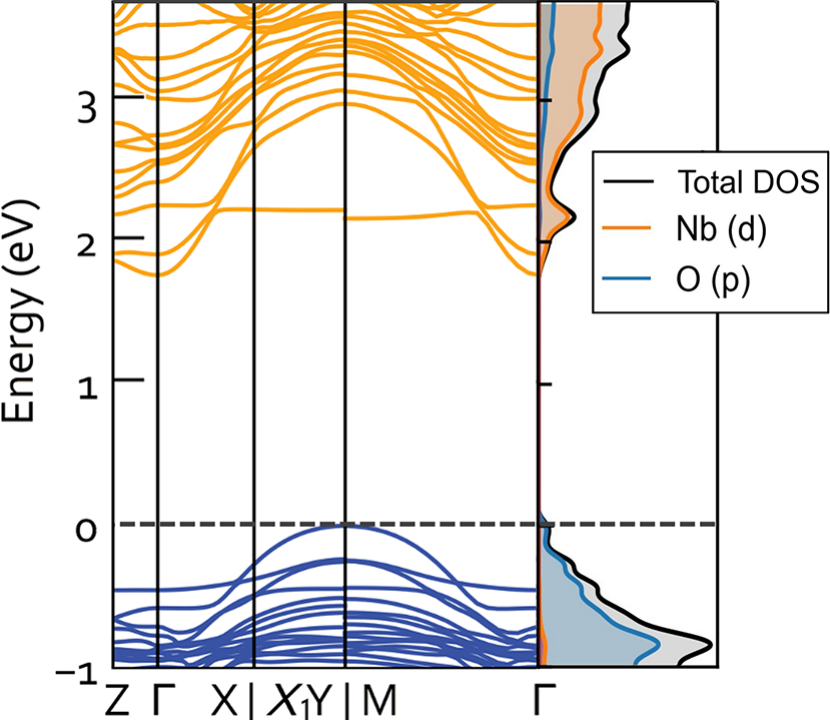
Band structure and density of states for NaNb_13_O_33_ calculated from the structure model from refinement of 1-NNO NPD obtained at 20 K. The Fermi level (dashed line) sits on top of the valence band.

Electronic structure calculations of the relaxed structure, starting from the model refined from 20 K neutron diffraction data, show a band diagram consisting of both flat and dispersive energy bands (Figure 4). Previous computational studies of similar Wadsley–Roth compounds have shown each *n* × *m* block capable of holding one localized electron in Nb d-orbitals dispersed over several octahedra.^26,72^ These orbitals form a relatively flat band in the band diagram because the corner-sharing octahedra are too far apart to allow for extensive electron delocalization. Conversely, overlapping orbitals between edge-sharing octahedra create dispersive conduction bands. When the number of electrons per block exceeds unity, these latter states allow electron delocalization along the *b*-axis via the shear planes.^20,21,73^ In the band structure of NaNb_13_O_33_, a relatively flat band is visible at the bottom of the conduction band (1.7–2.2 eV), indicating that electrons doping the structure from initial lithiation occupy such localized states as was seen with previous Wadsley–Roth compounds. With electron doping, the more dispersive bands beginning from 1.9 eV become accessible, suggesting electron delocalization along overlapping Nb d-orbitals and O p-orbitals in the shear planes. Experimental studies have demonstrated metallic conductivity along the shear planes when the one-electron-per-block threshold is exceeded.^18,29,74^ Thus, n-doping Wadsley–Roth phases with lithium insertion transform them from wide-band-gap insulators to good conductors. This insulator-to-metal transition as a function of lithiation has been observed at low lithium contents and obviates the need for nanostructuring or electron doping to achieve sufficient electronic conduction. From this band structure, we can therefore expect an insulator-to-metal transition and therefore good electronic conductivity as an electrode from NaNb_13_O_33_.

### Electrochemical Performance of NaNb_13_O_33_ in Li Cells

**Figure 5:**
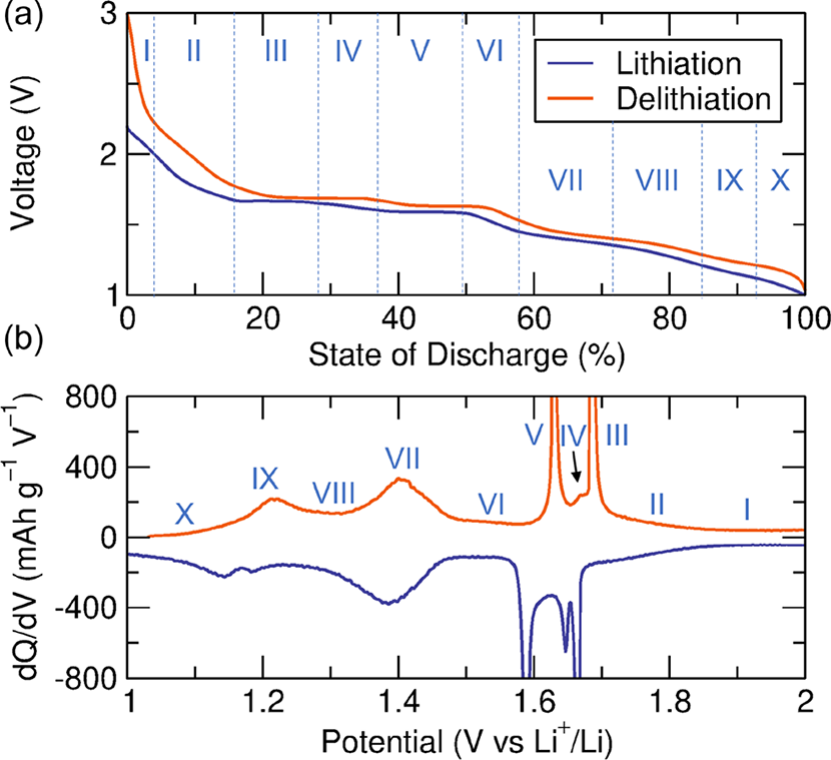
Galvanostatic charge/discharge (GCD) profiles and differential capacity analysis of a Li_*x*_NaNb_13_O_33_ vs Li cell after three C/15 formation cycles. (a) Galvanostatic profiles during lithiation and delithiation between 1 and 3 V vs Li^+^/Li at a rate of C/15. (b) Corresponding d*Q*/d*V* vs *V* plot for the GCD curves in (a). Regions in (a) and the corresponding features in (b) are labeled with Roman numerals. The cell had an areal active material mass loading of approximately 2 mg cm^–2^.

Galvanostatic cycling of 2-NNO after three formation cycles at C/15 is shown in Figure 5a. Both the lithiation and delithiation profiles exhibit at least ten different regions (labeled with Roman numerals), indicating a series of transitions between solid-solution and two-phase regions. To better identify these continuous and discontinuous phase transitions, we turned to differential capacity analysis (d*Q*/d*V* vs *V*). Figure 5b shows at least five peaks and four valleys. Each peak in the d*Q*/d*V* vs *V* plot suggests a phase transition that the active material undergoes during each lithiation and delithiation, with peaks in NaNb_13_O_33_ at 1.66 1.64, 1.58, 1.38, and 1.14 V vs Li^+^/Li. The overpotential and capacity offset between lithiation and delithiation are both small, indicating high energy efficiency and high Coulombic reversibility, respectively (Figure 5a).

**Figure 6:**
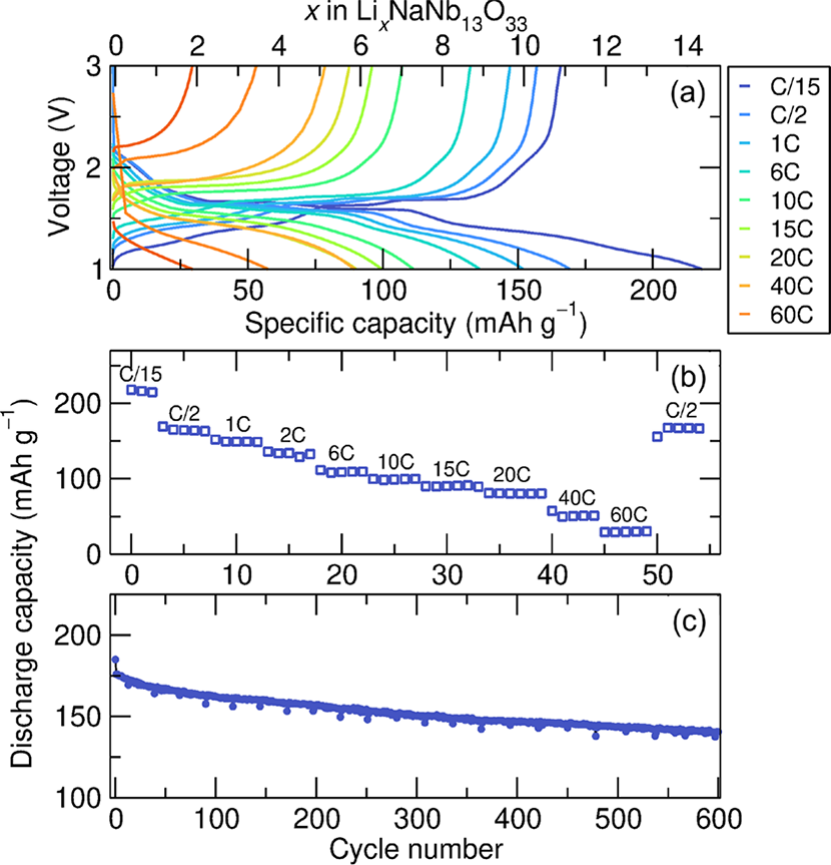
Rate performance of a Li_*x*_NaNb_13_O_33_//Li cell with an areal active material mass loading of 2 mg cm^–2^. (a) Representative galvanostatic charge–discharge curves at various C-rates after three formation cycles at C/15. (b) Specific capacities from (a) as a function of cycle number with increments in C-rate. (c) Specific capacity as a function of cycle number. Long-term cycling between 1 and 3 V was performed at a rate of 2C following two formation cycles at a rate of C/10.

Symmetric rate capability was also characterized in NaNb_13_O_33_ half-cells (Figure 6) after three formation cycles at C/15. At 6C and 20C current densities, NaNb_13_O_33_ delivered 108 and 80 mAh g^–1^ in approximately 5.4 and 1.2 min, respectively, indicating fast Li^+^ insertion and extraction despite primary particles on the order of 1–10 μm (Figure S4 inset). Furthermore, at higher active material mass loadings (5.5 mg cm^–2^), NaNb_13_O_33_ still exhibited a specific capacity of 95 mAh g^–1^ at 10 C (Figure S10). We note that the clear plateaus in the galvanostatic profiles and peaks in the d*Q*/d*V* curves are suppressed or merge at rates of 6C and above (Figure S11). The application of higher currents can lead to a nonequilibrium distribution of lithium through the electrode and/or individual particles, resulting in the coexistence of phases as indicated by the increasing width and decreasing height and area of the d*Q*/d*V* peaks (Figure S12). Increasing polarization with increasing current density is captured in the shifting d*Q*/d*V* peak locations. As a result, charge and discharge cutoff voltages are prematurely reached without achieving full removal or insertion of lithium (Figure S12a,e). These shifts in polarization are symmetric across lithiation and delithiation, indicating that ohmic and faradaic losses are similar during charge and discharge in this material.

Based on the above analysis, long-term cycling of lithium in NaNb_13_O_33_ half-cells from 1 to 3 V vs Li^+^/Li was conducted at a charge/discharge rate of 2C after two C/10 formation cycles. After 600 cycles, the material shows 80% capacity retention (Figure 6c). As the number of cycles increases, the electrochemical features are not as pronounced compared to those of the first few cycles (Figures S13 and S14). This suggests a material transformation such as decreased long-range order or increased heterogeneity upon cycling. We note that the cycled cells experienced periodic temperature fluctuations, which accounts for the outliers in discharge capacity. We also note that no attempts were made to optimize this electrochemical performance, although it could likely be further improved through, e.g., calendering, conductive carbon network optimization, or electrolyte optimization.

The state-of-the-art high-rate lithium-ion battery anode material is lithium titanate spinel Li_4_Ti_5_O_12_ (LTO). Based on a three-electron reduction to Li_7_Ti_5_O_12_, the gravimetric capacity of lithium titanate is 175.1 mAh g^–1^.^75^ However, bulk Li_4_Ti_5_O_12_ does not function well as an anode material and must be carbon-coated and nanoscaled to achieve high-rate (de)intercalation.^75,76^ These synthetic modifications add cost and complexity to the manufacturing process, while nanoparticles show decreased packing density and enhanced side reactions between the electrode particle surfaces and the electrolyte.^77^ Additionally, LTO contains a large quantity of inactive lithium, which is disadvantageous in terms of resource utilization and because the price of lithium has seen a multifold increase in recent years. Wadsley–Roth phases have become well-known for their ability to rapidly store large quantities of lithium (above 200 mAh g^–1^, beyond one Li per transition metal) even in micrometer-scale particles and in the absence of carbon coating.^18−20^ Generally, only 3/5 of the Ti^4+^ is reduced in Li_4_Ti_5_O_12_ while Wadsley–Roth phases see full one-electron or multielectron redox, leading to higher gravimetric and volumetric capacities in the latter phases. The initial results on bulk NaNb_13_O_33_ show that it is capable of lithium storage in excess of 200 mA h g^–1^ and retains about 50% capacity in a 5 min discharge relative to its 15 h discharge. Nonetheless, the maximum low-rate capacity and the high-rate capacity retention are lower than those observed in Wadsley–Roth phases such as TiNb_2_O_7_ and Nb_16_W_5_O_55_. Additional electrochemical testing under optimized and standardized conditions would be warranted to consider the commercial applicability of NaNb_13_O_33_ as a high-rate oxide anode. Synthetic modifications such as doping and/or carbon coating could be pursued to improve the electronic conductivity and enhance the rate performance by minimizing ohmic losses observed at high current densities (Figure 6a).

Electrochemical sodiation of 2-NNO electrodes was also attempted. NaNb_13_O_33_ half-cells vs Na metal were cycled at rates from C/10 to C/100. As predicted by variable-temperature NMR and BVSE mappings, little to no sodium intercalation was observed (Figure S15). The calculated sodium hopping barriers in related Wadsley–Roth phases are extremely high.^29^ It appears that the channel sizes and inflexibility of the framework are well-suited for rapid and reversible lithium intercalation but effectively hinder sodium conduction.

## Conclusion

Like many Wadsley–Roth phases, NaNb_13_O_33_ displays excellent lithium storage capacity that is largely maintained at high rates and over long-term cycling, even with micrometer-scale particles. Combined X-ray and neutron diffraction and ^23^Na solid-state NMR spectroscopy reveal that this Wadsley–Roth-like structure contains vacancies on the square-planar sodium site and that some sodium is present in the perovskite-like sites inside the 5 × 3 octahedral blocks of NaNb_13_O_33_. Reduction of Nb from the 5+ to the 4+ and partially to the 3+ oxidation state enables large lithium capacities, which do not seem inhibited by the presence of Na point defects in the block interiors, although the precise effects of this Na disorder on Li conduction were not investigated. Future studies might attempt to minimize the extent of this disorder, perhaps through lower-temperature synthesis techniques, and this should be easily quantifiable with ^23^Na solid-state NMR. Bond valence mapping suggests fast, quasi-1D Li diffusion down parallel block channels and facile hopping between neighboring channels, which explains the excellent rate performance observed (e.g., 100 mA h g^–1^ at 20 C) despite the presence of tunnel blockages and large particle sizes. DFT electronic structure calculations are consistent with previous studies and suggest that there will be good electronic conductivity primarily along overlapping niobium d-orbitals and oxygen p-orbitals within shear planes once NaNb_13_O_33_ is n-doped by Li insertion. Intercalation of Na was also tested, but high Na hopping barriers prevented any significant electrochemical energy storage, in agreement with BVSE calculations. Altogether, this work provides structural and electrochemical insights into a promising new high-rate lithium-ion battery anode material and expands the use of Wadsley–Roth battery materials into new compositional and structural phase space.

## Supporting Information Available

X-ray and neutron diffraction Rietveld fits, nuclear magnetic resonance spectra and scanning electron micrographs of pristine NaNb_13_O_33_ samples, X-ray diffraction of NaNbO_3_ used for NMR analysis, NMR and X-ray diffraction supporting phase mapping, variable-temperature ^23^Na NMR spectra, Fourier difference map of NaNb_13_O_33_, and additional galvanostatic cycling data, including representative d*Q*/d*V* curves and analysis and sodium half-cell cycling (PDF)
Crystallographic information file of Na*_x_*Nb_13_O_33_ at 300 K (NB: sodium occupancies were refined freely to test the model stability and thus do not sum precisely to NaNb_13_O_33_; see main text for details) (CIF)
Crystallographic information file of Na_*x*_Nb_13_O_33_ at 20 K (NB: sodium occupancies were refined freely to test the model stability and thus do not sum precisely to NaNb_13_O_33_; see main text for details) (CIF)

## Supplementary Material

cm3c01066_si_001.pdf

cm3c01066_si_002.cif

cm3c01066_si_003.cif

## Notes

*Note*: Several days before the submission of this manuscript, a paper appeared that focused on the electrochemical performance of NaNb_13_O_33_.^78^ In this work, the room-temperature and low-temperature performance of NaNb_13_O_33_ are studied in detail. The applied focus of that work differs from the fundamental crystal chemical questions addressed through combined high-resolution diffraction and solid-state NMR in the present study.
