# Supplementary material for: Rapid and Reversible Lithium Insertion in the Wadsley–Roth-Derived Phase NaNb13O33
Source: Chem Mater. 2023 Aug 2;35(16):6364–73. doi: 10.1021/acs.chemmater.3c01066 (PMC10449011; doi:10.1021/acs.chemmater.3c01066)
Supplement: Supplementary file 1 — cm3c01066_si_001.pdf [file cm3c01066_si_001.pdf]

Rapid and Reversible Lithium Insertion in the Wadsley–Roth-derived Phase $NaNb_{13}O_{33}$

Ashlea R. Patterson,[†] Rodrigo Elizalde-Segovia,[‡] Kira E. Wyckoff,[†] Arava Zohar,[†] Patrick P. Ding,[¶] Wiley M. Turner,[¶] Kenneth R. Poeppelmeier,[¶] Sri R. Narayan,[‡] Raphäele J. Clément,[†] Ram Seshadri,[†] and Kent J. Griffith[*,¶,§,‖]

†*Materials Department and Materials Research Laboratory*

University of California Santa Barbara, California 93106, United States

‡*Department of Chemistry, University of Southern California*

Los Angeles, California 90007, United States

¶*Department of Chemistry, Northwestern University*

Evanston, IL, 60208, United States

§*Department of Materials Science and Engineering, Northwestern University*

Evanston, IL, 60208, United States

‖*Present address: Department of Chemistry and Biochemistry, University of California*

San Diego, La Jolla, CA, 92093, United States

E-mail: k3griffith@ucsd.edu

Structural refinements. The background for NPD datasets from Bank 2 and Bank 3 and SXRPD fit with Chebyshev polynomials with 9, 6, and 12 terms, respectively. Niobium and oxygen occupancies were fixed. The background, lattice parameters, SXRPD profile parameters, scale factor, Na occupancy, and isotropic atomic displacement parameters were all refined during analysis. N.b. Na1 occupancy was refined freely and there are vacancies on this site owing to the presence of sodium within the blocks as explained in the main text, so the formula from the crystallographic data deviates from $NaNb_{13}O_{33}$.

Table S1. Structural refinement of 1-NNO from joint synchrotron X-ray diffraction and neutron powder diffraction.

Space group No. 5 (C12/m1)

T = 300 K

a = 22.40697(5) Å

b = 3.834022(6) Å

c = 15.36263(3) Å

β = 91.42669(18) °

V = 1319.376(3) Å^{-3}

R_F = 0.03248 wR_F = 0.05451 GoF = 4.30

Atom	x	y	z	Occ.	Ui_{iso}
Na1	0.00000	0.50000	0.50000	0.89(2)	0.0462(23)
Nb1	0.00000	0.00000	0.00000	1.0000	0.0130(6)
Nb2	0.06966(9)	0.00000	0.22976(14)	1.0000	0.0118(4)
Nb3	0.22555(7)	0.00000	0.13615(11)	1.0000	0.00457(31)
Nb4	0.15531(7)	0.00000	0.90276(11)	1.0000	0.00394(31)
Nb5	0.13738(6)	0.00000	0.46756(11)	1.0000	0.00680(30)
Nb6	0.29352(6)	0.00000	0.37418(10)	1.0000	0.00353(26)
Nb7	0.08609(6)	0.00000	0.67337(10)	1.0000	0.00415(28)
O1	0.08167(12)	0.00000	0.94984(19)	1.0000	0.0111(4)
O2	0.00000	0.50000	0.00000	1.0000	0.0130(7)
O3	0.32066(10)	0.00000	0.09937(15)	1.0000	0.0042(4)
O4	0.25000(9)	0.00000	0.86249(14)	1.0000	0.0056(4)
O5	0.15410(10)	0.00000	0.19521(16)	1.0000	0.0101(5)
O6	0.42998(10)	0.00000	0.75390(14)	1.0000	0.0115(4)
O7	0.19418(10)	0.00000	0.02673(16)	1.0000	0.0108(4)
O8	0.03511(13)	0.00000	0.12112(21)	1.0000	0.0103(4)
O9	0.12920(10)	0.00000	0.78550(14)	1.0000	0.0063(4)
O10	0.26851(10)	0.00000	0.26416(15)	1.0000	0.0084(4)
O11	0.09920(11)	0.00000	0.36416(16)	1.0000	0.0137(5)
O12	0.01166(11)	0.00000	0.70993(16)	1.0000	0.0116(5)
O13	0.39128(9)	0.00000	0.33573(14)	1.0000	0.0076(4)
O14	0.18077(9)	0.00000	0.61084(14)	1.0000	0.0063(4)
O15	0.22370(9)	0.00000	0.43189(14)	1.0000	0.0076(4)
O16	0.34753(8)	0.00000	0.50429(12)	1.0000	0.00332(30)
O17	0.06808(8)	0.00000	0.54471(13)	1.0000	0.0063(4)

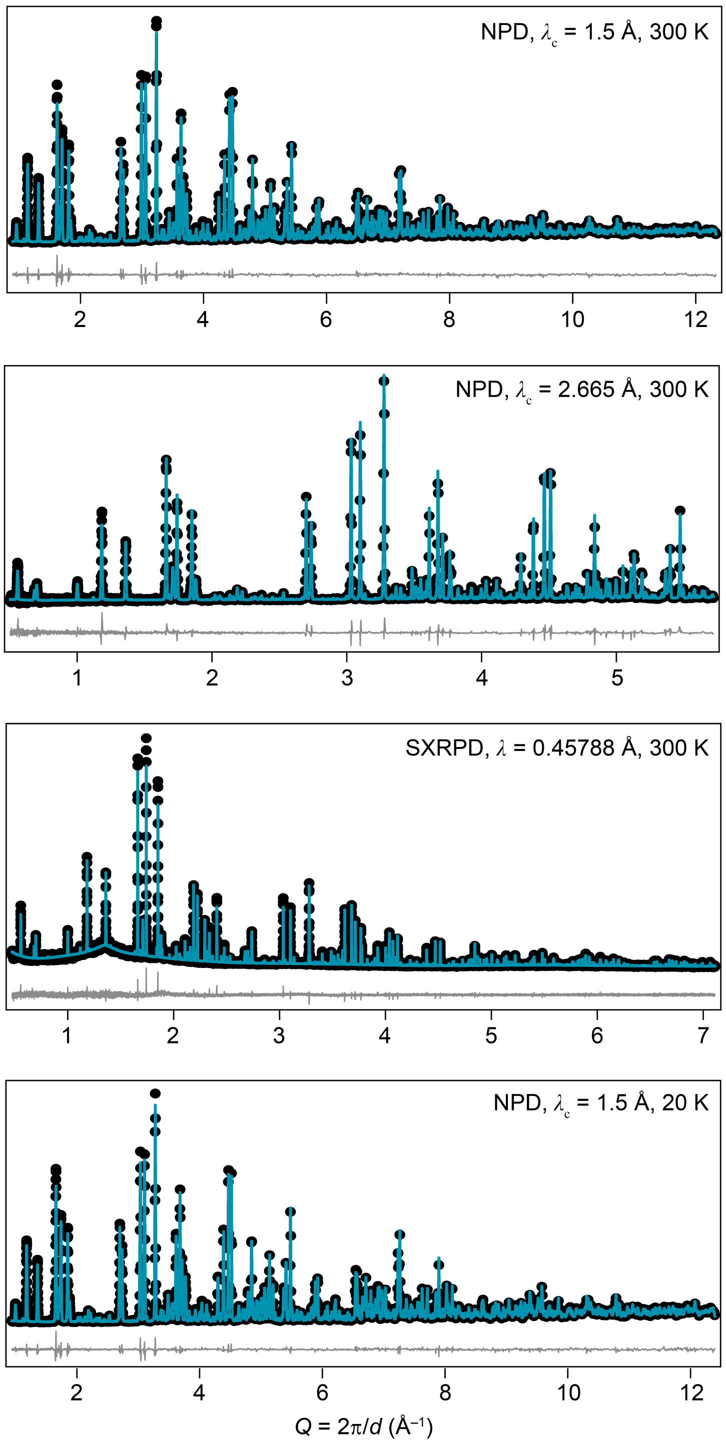

Figure S1: Time-of-flight neutron powder diffraction (TOF-NPD) data of 1-NNO obtained on the POWGEN diffractometer at the Spallation Neutron Source (SNS) located at Oak Ridge National Laboratory, and synchrotron X-ray powder diffraction data of 1-NNO collected on the 11-BM beamline at the Advanced Photon Source at Argonne National Laboratory.

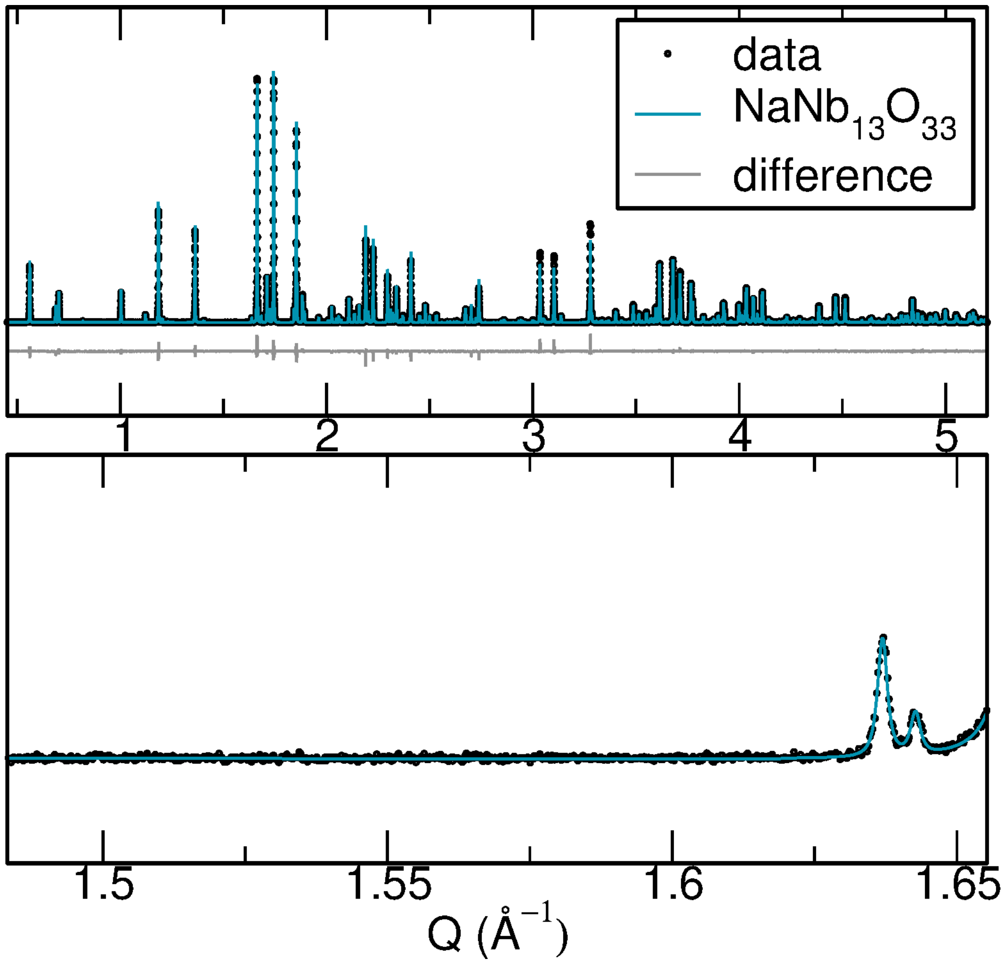

Figure S2: Top: Synchrotron X-ray diffraction of 2-NNO collected on the 11-BM beamline at the Advanced Photon Source at Argonne National Laboratory. Bottom: Zoom of the region in which the common $Na_{13}Nb_{35}O_{94}$ impurity phase would result in a peak around 1.55 Å^{-1}, showing the absence of this impurity phase.

NMR of 2-NNO. A solid-state ^{23}Na MAS NMR spectrum of the 2-NNO material was acquired using a Bruker AVANCE III Ultrashield Plus 800 MHz (18.8 T) spectrometer and a 3.2 mm Bruker HX MAS probe. The 2-NNO sample was packed into a 3.2 mm zirconia rotor in air and spun about the magic angle at 20 kHz. The 2-NNO Hahn echo spectrum was collected with an optimized recycle delay of 0.8 seconds, and referenced against a 1 M aqueous solution of NaF. NMR spectra were processed in Bruker TopSpin and DMFit.[1]

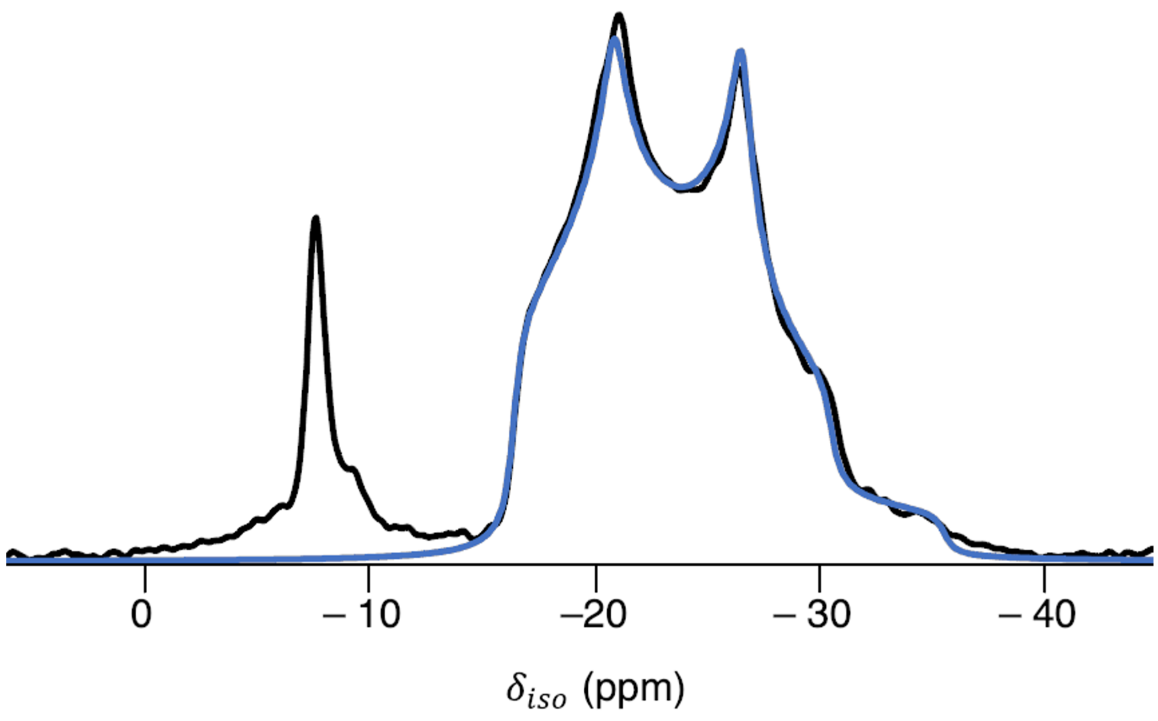

Figure S3: Room-temperature ^{23}Na MAS NMR spectrum of 2-NNO showing two distinct resonances (black line), a fit of the quadrupolar environment centered at approximately −24 ppm (blue line), which corresponds to Na in its expected square-planar environment in $NaNb_{13}O_{33}$ and a sharper resonance at approximately –8 ppm. This sharp resonance corresponds to Na occupying the perovskite-like sites within the octahedral blocks. The apparent difference in shift of the square-planar sodium site at 9.4 T and 18.8 T results from the quadrupolar shift contribution.

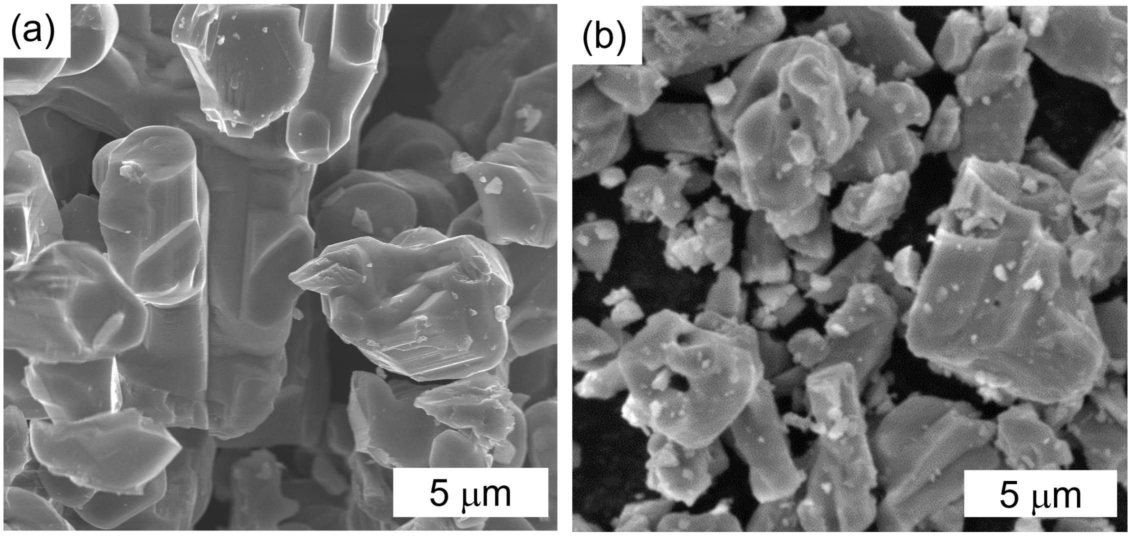

Figure S4: Comparison of (a) 1-NNO and (b) 2-NNO particle sizes via scanning electron microscopy. Electrochemical measurements were performed on 2-NNO electrodes.

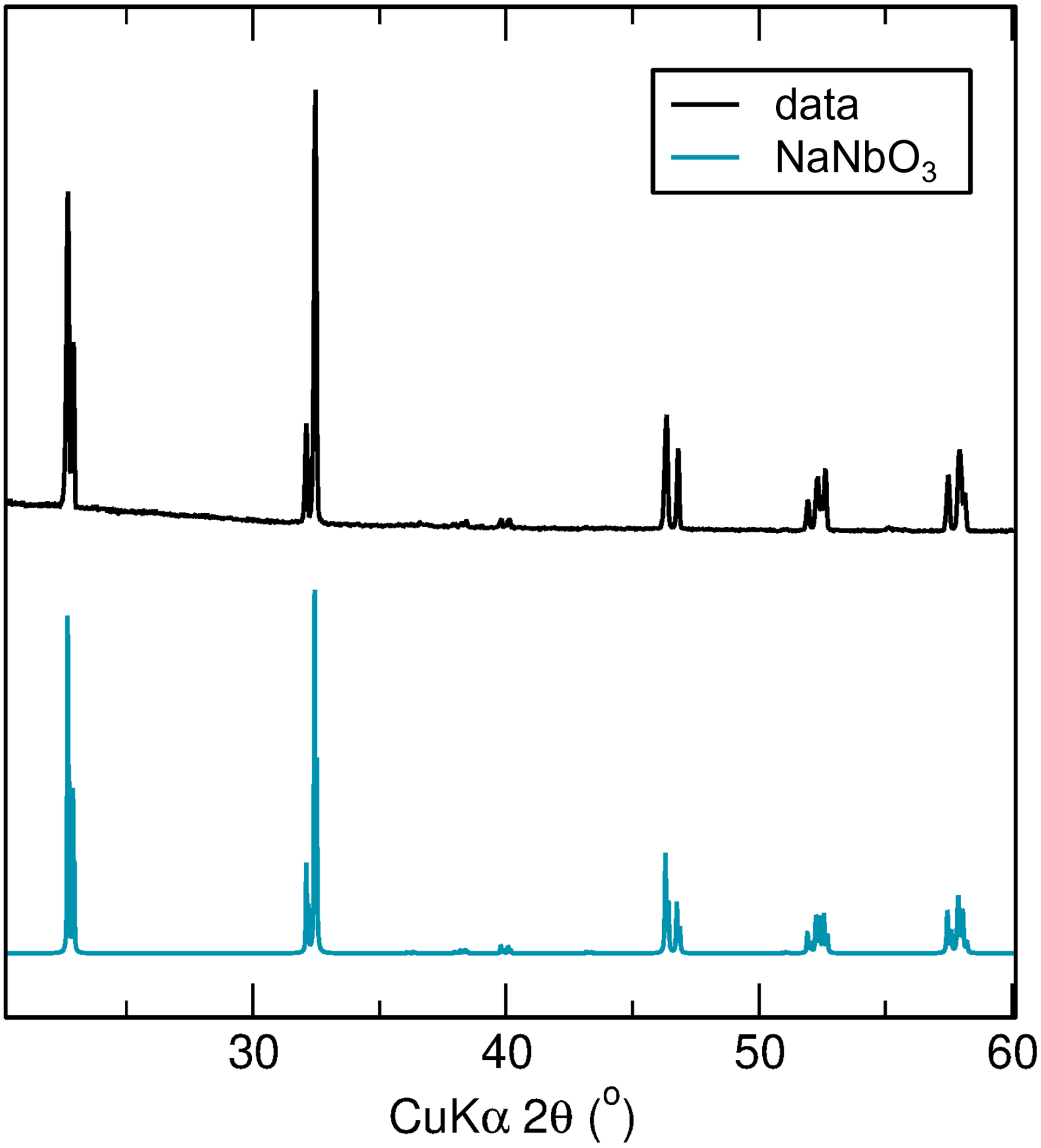

Figure S5: Laboratory X-ray diffraction of as-prepared $NaNbO_3$ (top) and a simulated reference pattern from ICSD 247311 (bottom).[2]

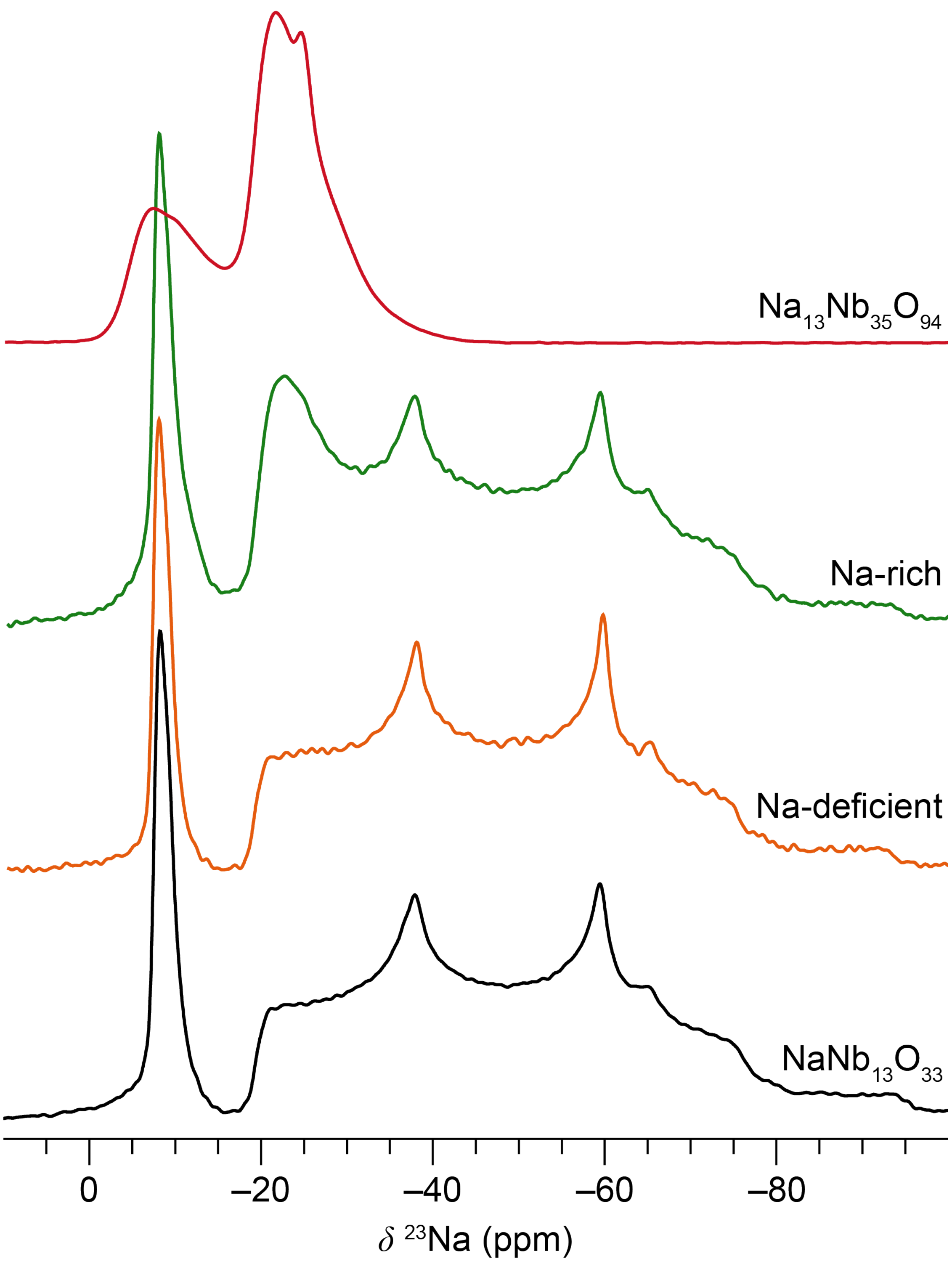

Figure S6: ^{23}Na NMR spectra of $Na_{13}Nb_{35}O_{94}$ as well as stoichiometric, Na-rich, and Na-deficient samples of $NaNb_{13}O_{33}$ recorded at 9.4 T and 12.5 kHz MAS. Na-rich and Na-deficient samples were prepared with excess Na_2CO_3 and Nb_2O_5, respectively.

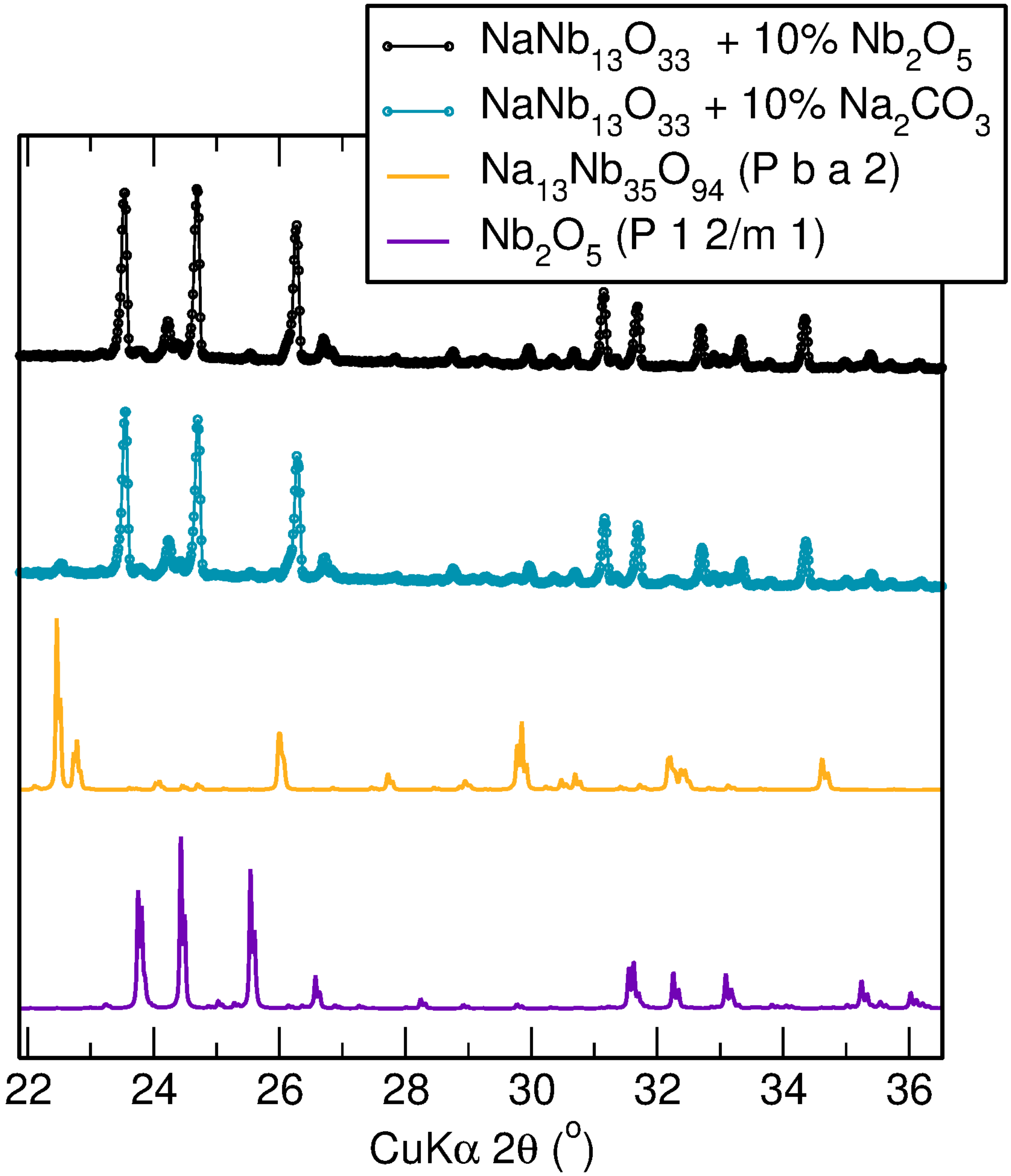

Figure S7: Laboratory X-ray diffraction patterns of as-prepared $Na_{13}Nb_{35}O_{94}$ as well as Na-rich and Na-deficient $NaNb_{13}O_{33}$. Minor $Na_{13}Nb_{35}O_{94}$ and Nb_2O_5 secondary phases are visible in the diffraction patterns of Na-rich and Na-deficient $NaNb_{13}O_{33}$.

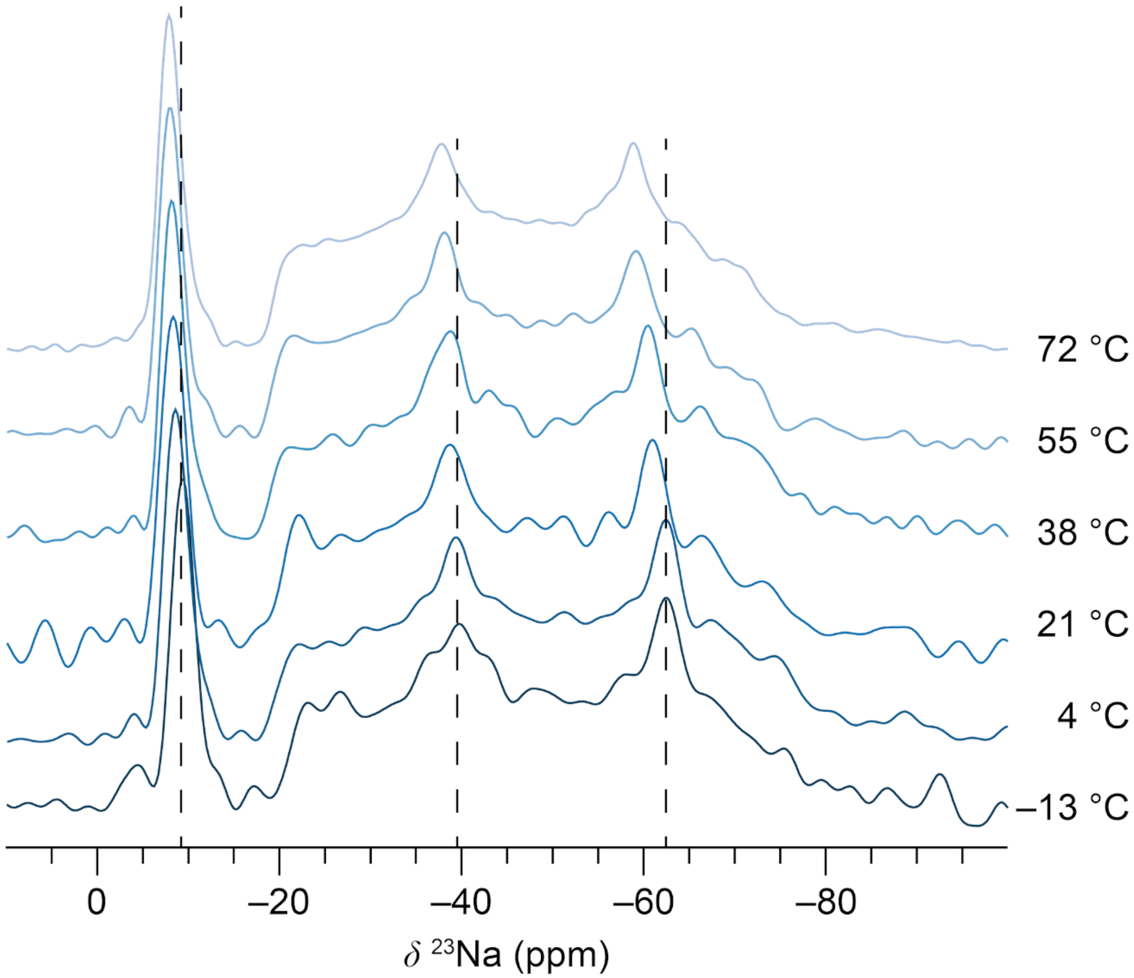

Figure S8: Variable-temperature ^{23}Na NMR spectra of $NaNb_{13}O_{33}$ collected at 9.4 T and 38 kHz MAS. Dashed lines are guides for the eye to see the small shift of all signals to higher frequency as a function of temperature.

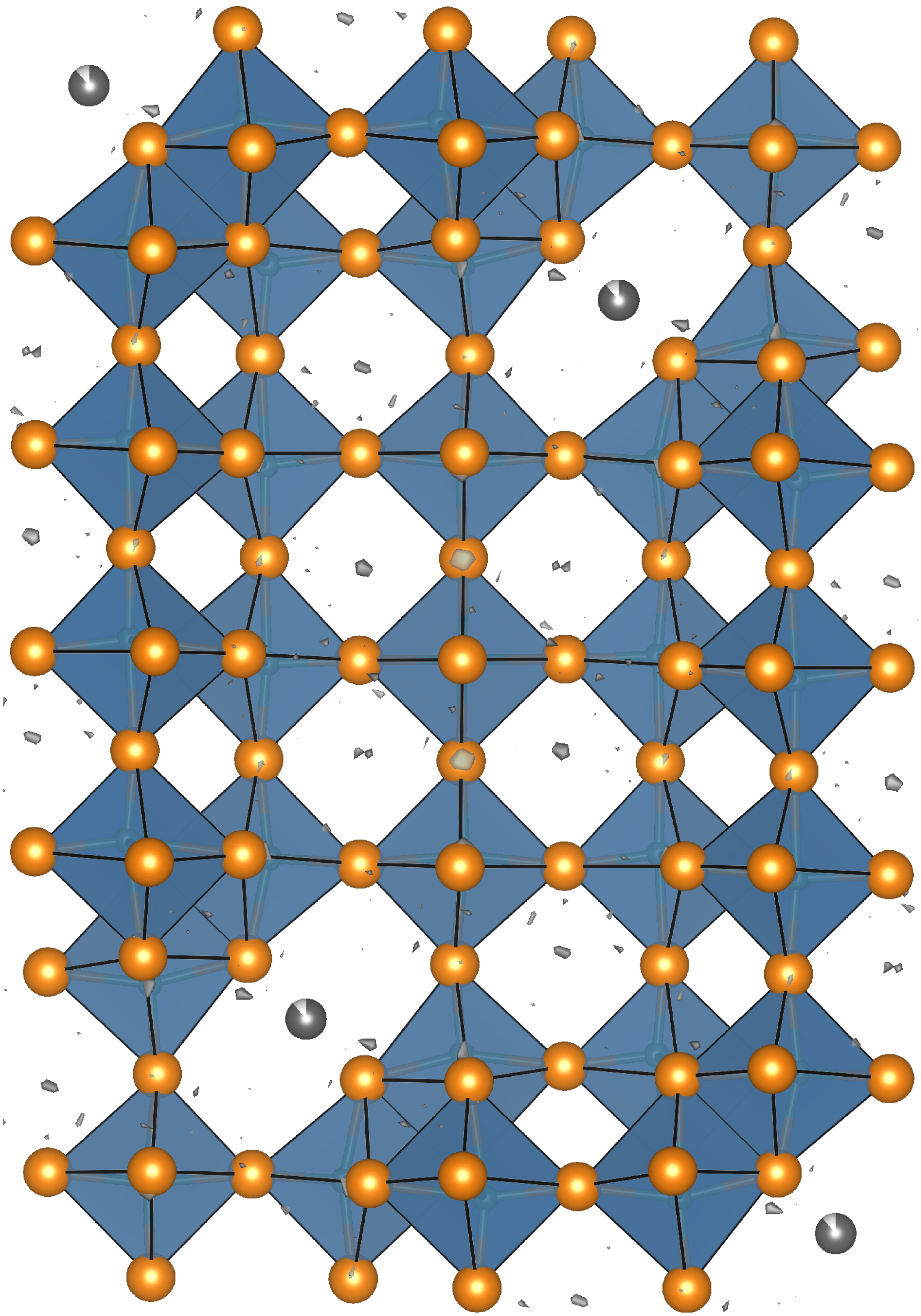

Figure S9: Fourier difference map showing small but finite Na density in A-site perovskite-like positions within $NaNb_{13}O_{33}$. Map produced from neutron diffraction data collected at 20 K.

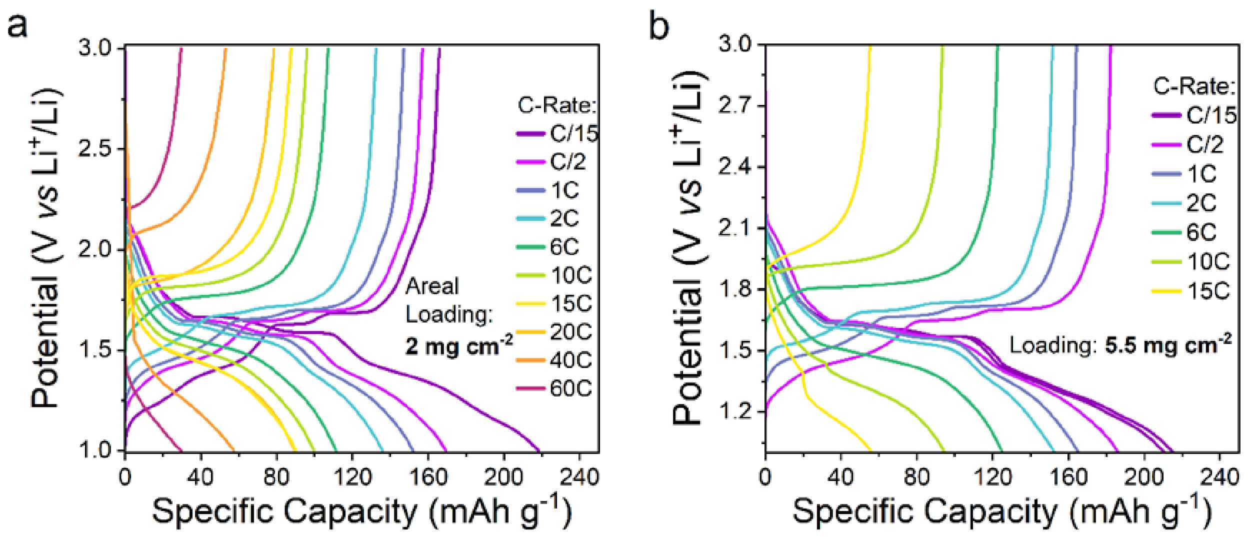

Figure S10: Representative galvanostatic charge–discharge curves at various C-rates for $NaNb_{13}O_{33}$//Li cells with an areal active material mass loading of a) 2.0 mg cm^{-2} and b) 5.5 mg cm^{-2}.

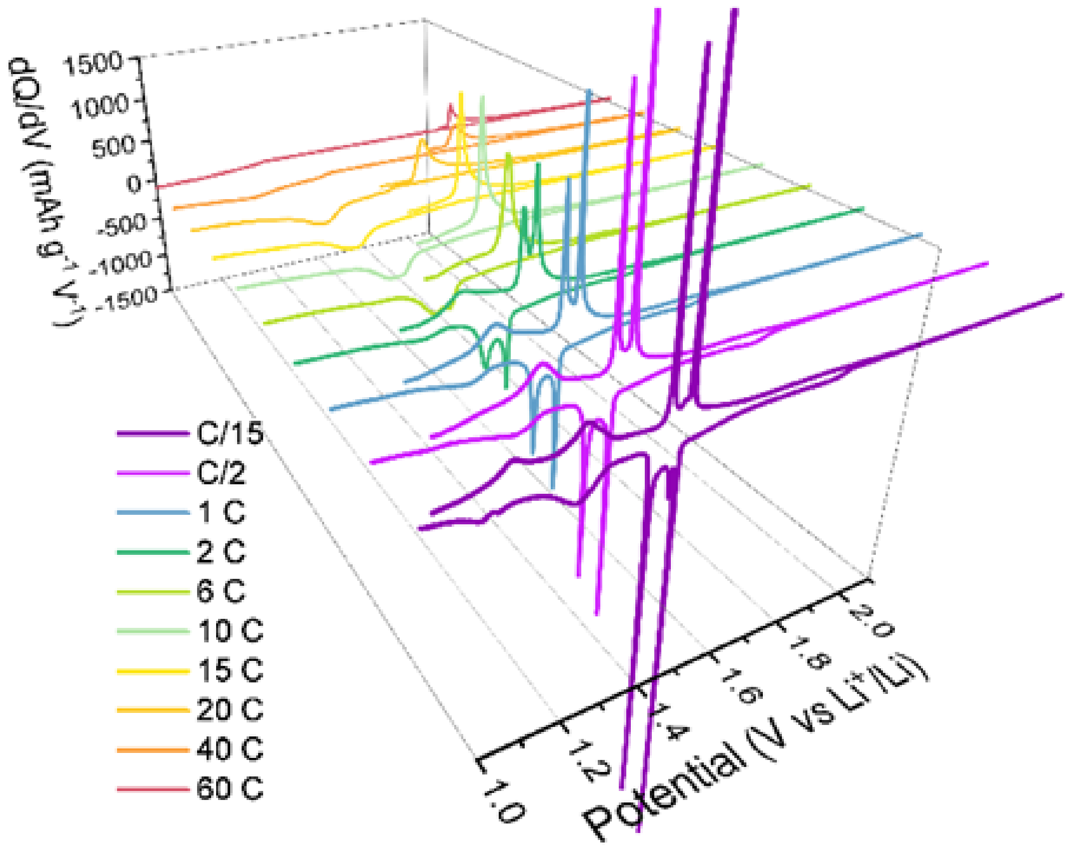

Figure S11: Three-dimensional dQ/dV plots of a $NaNb_{13}O_{33}$//Li cell from C/15 to 60C from the galvanostatic discharge curves shown in Figure SS10. The loss in dQ/dV peak definition at higher current densities is apparent.

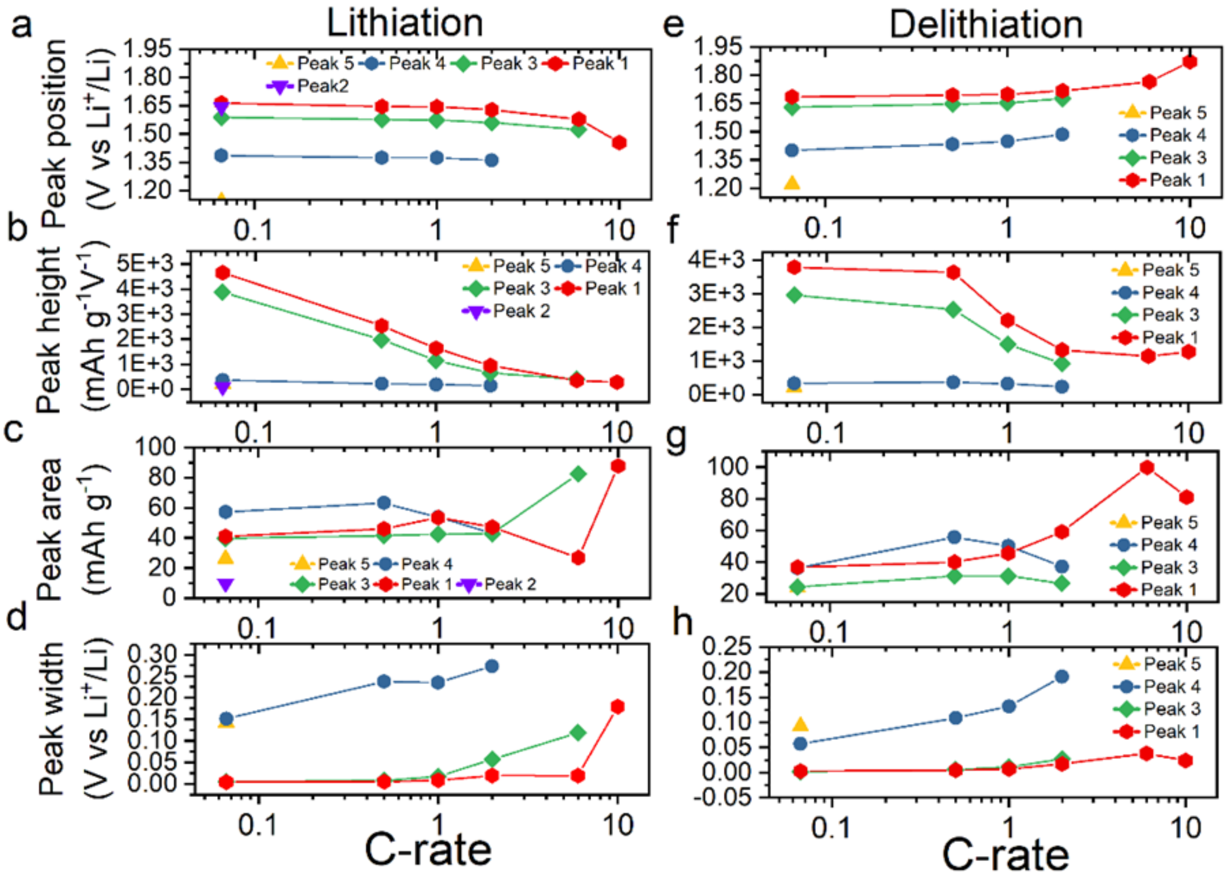

Figure S12: $NaNb_{13}O_{33}$//Li cell peak analysis as a function of C-rate during lithiation. a) Peak position, b) peak height, c) peak area, d) peak width.

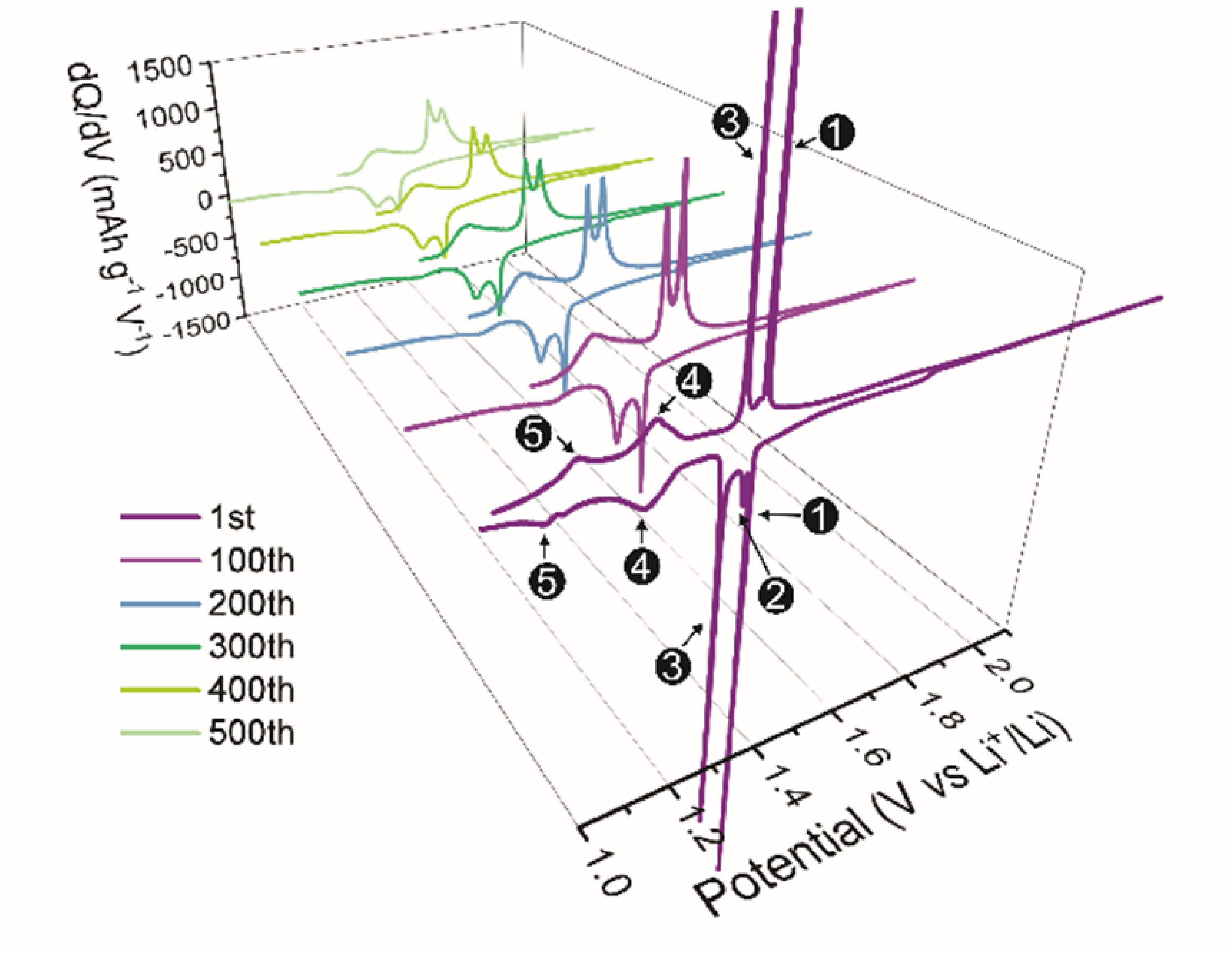

Figure S13: $NaNb_{13}O_{33}$//Li cell differential capacity analysis. Three-dimensional dQ/dV plots from the galvanostatic charge/discharge curves in Figure S14 taken from long-term cycling between 1 and 3 V vs Li^+/Li at 2C. The loss in dQ/dV peak definition over long-term cycling is apparent.

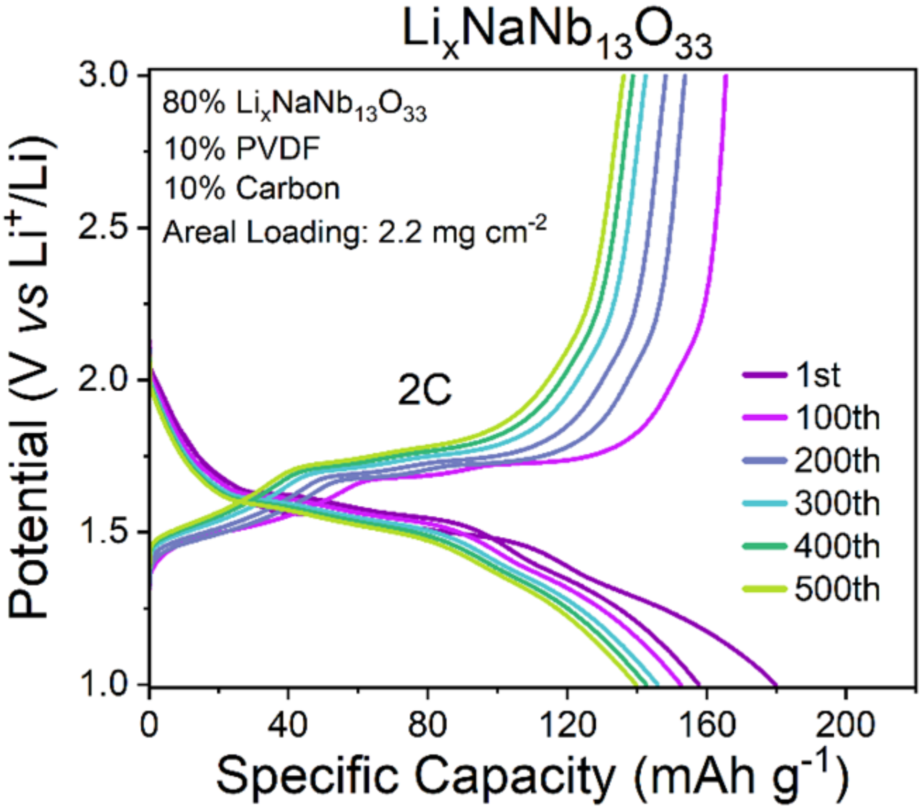

Figure S14: $NaNb_{13}O_{33}$//Li cell galvanostatic charge/discharge curves for the 1st, 100th, 200th, 300th, 400th and 500th cycles for an areal active-material mass loading of 2.2 mg cm $^{-2}$ under long-term cycling between 1 and 3 V vs Li^+/Li at 2C.

Fabrication of 2-NNO–Na half-cells. Half-cells of 2-NNO electrodes vs. sodium metal were fabricated with electrodes containing an 80:10:10 weight fraction of active material, conductive carbon, and binder. 2-NNO was ball-milled with carbon black (TIMCAL SuperP) for 30 minutes, and then combined with PVDF dissolved in NMP using a FlakTek speed mixer to form a slurry. The slurry was cast on polished copper foil with a doctor blade for a 120 μm thick electrode film, which was then dried under vacuum at 100 °C overnight and punched into 6 mm diameter disks with mass loading densities of 2 to 3 mg cm^{-2}. Swagelok cells were assembled in an Ar-filled glovebox with sodium metal foil as a counter electrode and a glass fiber separator (Whatman GF/D). The electrolyte was prepared by dissolving 1.0 M $NaPF_6$ ($\geq$99%, Strem Chemicals) in EC:DMC (1:1 w/w, $\geq$99%, Sigma-Aldrich).

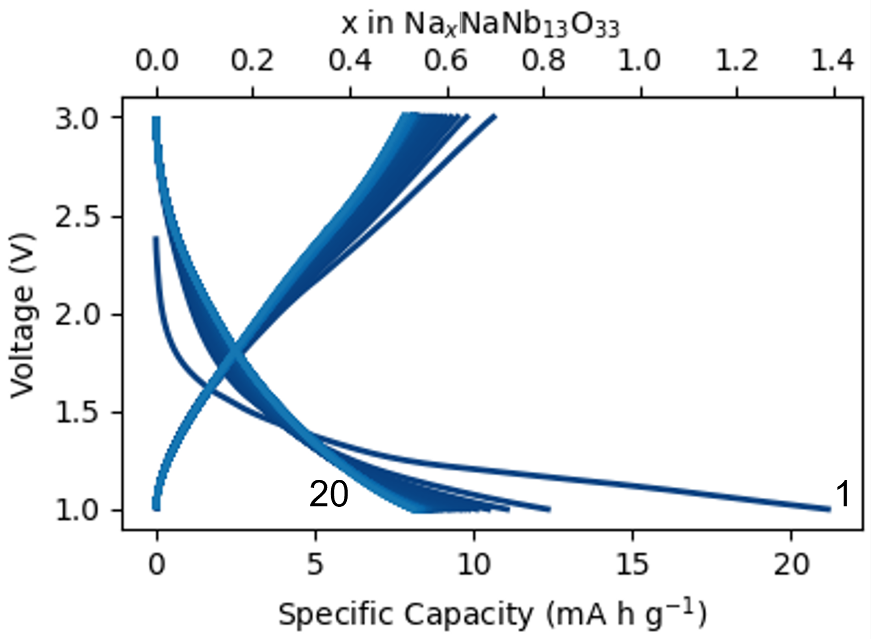

Figure S15: $NaNb_{13}O_{33}$//Na cell cycled between 1 V and 3 V vs Na^+/Na at C/60 rate. Cycle numbers 1 and 20 are denoted by their respective curves.

References

(1) Massiot, D.; Fayon, F.; Capron, M.; King, I.; Le Calvé, S.; Alonso, B.; Durand, J.-O.; Bujoli, B.; Gan, Z.; Hoatson, G. Modelling One-and Two-Dimensional Solid-State NMR Spectra. *Magn. Reson. Chem.* **2002**, *40*, 70–76.

(2) Johnston, K. E.; Tang, C. C.; Parker, J. E.; Knight, K. S.; Lightfoot, P.; Ashbrook, S. E. The Polar Phase of $NaNbO_3$: A Combined Study by Powder Diffraction, Solid-State NMR, and First-Principles Calculations. *J. Am. Chem. Soc.* **2010**, *132*, 8732–8746.
